# Deciphering Microbial Community Dynamics and Biochemical Changes During Nyons Black Olive Natural Fermentations

**DOI:** 10.3389/fmicb.2020.586614

**Published:** 2020-10-08

**Authors:** Marine Penland, Stéphanie-Marie Deutsch, Hélène Falentin, Audrey Pawtowski, Elisabeth Poirier, Giorgia Visenti, Christophe Le Meur, Marie-Bernadette Maillard, Anne Thierry, Jérôme Mounier, Monika Coton

**Affiliations:** ^1^Univ Brest, Laboratoire Universitaire de Biodiversité et Ecologie Microbienne, Plouzané, France; ^2^STLO, INRAE, Institut Agro, Rennes, France

**Keywords:** microbial dynamics and diversity, volatiles, metabarcoding, phenolic compounds, spontaneous fermentaion

## Abstract

French PDO Nyons black table olives are produced according to a traditional slow spontaneous fermentation in brine. The manufacture and unique sensorial properties of these olives thus only rely on the autochthonous complex microbiota. This study aimed at unraveling the microbial communities and dynamics of Nyons olives during a 1.5-year-long spontaneous fermentation to determine the main microbial drivers and link microbial species to key metabolites. Fermentations were monitored at a local producer plant at regular time intervals for two harvests and two olive types (organically and conventionally grown) using culture-dependent and metabarcoding (ITS2 for fungi, V3-V4 region for bacteria) approaches. Olives and brines were also sampled for volatiles, organic acids and phenolic compounds. No major differences in microbiota composition were observed according to olive type or harvest period. Throughout the fermentation, yeasts were clearly the most dominant. ITS2 sequencing data revealed complex fungal diversity dominated by *Citeromyces nyonsensis*, *Wickerhamomyces anomalus*, *Zygotorulaspora mrakii*, *Candida boidinii* and *Pichia membranifaciens* species. Bacterial communities were dominated by the *Celerinatantimonas* genus, while lactic acid bacteria remained scarce. Clear shifts in microbial communities and biochemical profiles were observed during fermentation and, by correlating metabolites and microbiota changes, four different phases were distinguished. During the first 7 days, phase I, a fast decrease of filamentous fungal and bacterial populations was observed. Between days 21 and 120, phase II, *W. anomalus* and *C. nyonsensis* for fungi and *Celerinatantimonas diazotrophica* for bacteria dominated the fermentation and were linked to the pH decrease and citric acid production. Phase III, between 120 and 183 days, was characterized by an increase in acids and esters and correlated to increased abundances of *Z. mrakii*, *P. membranifaciens* and *C. boidinii.* During the last months of fermentation, phase IV, microbial communities were dominated by *P. membranifaciens* and *C. boidinii*. Both species were strongly correlated to an increase in fruity esters and alcohol abundances. Overall, this study provides an in-depth understanding about microbial species succession and how the microbiota shapes the final distinct olive characteristics. It also constitutes a first step to identify key drivers of this fermentation.

## Introduction

Table olives are among the most consumed fermented vegetables with 2.57 million tons in 2019 (International Olive Council, 2019). They originate from the Mediterranean area and are produced and consumed worldwide. Table olives are appreciated by consumers for their characteristic taste and pleasant aromas. Moreover, many health and nutritional benefits have been reported as they are rich in polyunsaturated fatty acids and polyphenols (Conte et al., 2020).

Initially, a fermentation step was mandatory to reduce the natural bitterness of the fruits and make them edible. Different preparation methods have been reported depending on traditional know-how, olive variety and maturity, although three main methods are used nowadays (Romeo, 2012). For Spanish-style green olives, debittering is performed in 1.6–2.3% lye depending on the variety and harvest time prior to fermentation in 6–8% salt brine. The Californian-style olive preparation includes lye treatment after brining and fruit darkening by chemical or heat treatment. Finally, for Greek-style black olives, the debittering process occurs during natural fermentations in brine with a 6–10% salt concentration.

Regardless of the method, fermentation is carried out by diversified, often autochthonous, microbial communities. Lactic acid bacteria and yeasts, naturally present on olives, are considered to be the main drivers of the process and produce diverse organic acids among other metabolites (Randazzo et al., 2017). Different studies have described the microbial diversity encountered in some olive fermentations, especially for Spanish- and Greek-style olives from *Manzanilla* and *Alorena de Malaga* varieties. The most frequently described genera are *Lactiplantibacillus* (ex-*Lactobacillus*) and *Leuconostoc* for bacteria and *Pichia*, *Candida*, *Wickerhamomyces* and *Saccharomyces* for yeasts (Hurtado et al., 2012; Heperkan, 2013). In addition, intensive studies on the microbial diversity encountered in different varieties have been conducted and revealed that the indigenous microbiota was strongly influenced by olive cultivar and maturity (Hurtado et al., 2009) and preparation parameters, such as temperature and salt concentration. This directly impacts table olive characteristics as microbial communities also induce chemical changes in olives. Thus, olive flavor is closely related to qualitative and quantitative volatile and non-volatile acids (Panagou and Tassou, 2006). In spite of this, few studies have investigated the volatile profile of fermented olives and the relation to microbial communities throughout the fermentation process except for green olive fermentations.

Nyons table olives are distinct for many reasons. It is one of the table olive types granted PDO in southern France. They are well-known for their dark brown color, their melting texture and typical flowery aromas. The traditional fermentation process follows specific requirements: Tanche variety olives must be harvested at full maturity during the winter season (mid-December to early February) and directly submerged in brine where they undergo spontaneous fermentation without microbial adjuncts. From a technical perspective, one specific and interesting aspect of this process is that fermentation can last up to 1.5 years. Current knowledge about the microbial diversity of Nyons table olives is limited to yeast diversity and dynamics, as assessed by a culture-dependent approach (Coton et al., 2006). The data obtained from this study revealed rich yeast diversity and led to the identification of two new species, *Citeromyces nyonsensis* (Casaregola et al., 2013) and *Saccharomycopsis olivae* (Jacques et al., 2014), however, bacterial communities and detailed microbial dynamics were not described.

Thus, the objective of this study was to unravel the microbial dynamics during natural olive fermentations, using conventionally and organically grown Nyons olives, and to determine to what extent they contribute to the final product characteristics. For this purpose, the strategy was to (i) identify the microorganisms involved in Nyons table olive fermentations and understand their dynamics by culture-dependent and metabarcoding analyses, (ii) assess the impact of olive maturity and cultivation practices (organic or conventional olives) on the fermentation process and (iii) investigate changes in the biochemical profile during fermentation to better understand the link between microorganisms and organoleptic characteristics of these olives.

## Materials and Methods

### Olive Fermentation Preparation and Sampling

Samples were obtained from PDO Nyons table olive fermentations directly from the Vignolis cooperative located in Nyons, France. Four different fermentations were monitored over a 1.5-year period between January 2018 and May 2019. First, to investigate the impact of olive fruit maturity on fermentation, two harvests were studied: one harvest started in early January (R1) while the second harvest was in late January (R2). Secondly, to investigate the impact of agricultural practices on the fermentation process, both organic and conventional olives were separately used for both harvests. For each fermentation condition, named R1-Orga, R1-Conv, R2-Orga and R2-Conv, three independent fermentation tanks were prepared for each condition using the same olive batch (biological replicates). Tanks were prepared at the cooperative facilities and according to the traditional PDO process as follows: 300 kg of freshly harvested and lightly water washed olives were submerged in 200 L of cover brine (10% salt) and then sealed with a heavy lid to limit the air layer. Then, tanks were left untouched, except for sampling, during fermentation in the cooperative storage area and at room temperature (<18°C). Brine and olive samples were taken at days 1 (fresh fruits), 8, 21, 42, 64, 120, 183, 267, and ∼480 (endpoint), stored between 12 and 16°C, and processed within 24 h.

### Microbial Counts During Nyons Olive Fermentations

Microbial populations of interest were monitored during fermentation for each tank. Brine and olive fruits were treated together in a 1:1 ratio (w/w): 12.5 g of olive flesh and 12.5 mL of brine were mixed with 225 mL buffered peptone water and blended with a stomacher for 3 min at high speed. Serial dilutions were prepared in Tryptone Salt diluent (TS; sodium chloride 8.5 g/L, tryptone 1 g/L) and plated on seven different media to enumerate: total fungal populations (Yeast extract Glucose Chloramphenicol medium (YGC, 25°C, 5 days), halotolerant fungi (YGC+5 % NaCl, 25°C, 5 days), total microbial populations on Plate Count Agar (PCA, 30°C, 72 h), halotolerant microbial populations on PCA+5% NaCl (30°C, 72 h), enterobacteria on Violet Red Bile Glucose agar (VRBG, 30°C, 48 h), lactic acid bacteria (LAB) on de Man Rogosa Sharpe agar (MRS+0.01% cycloheximide to prevent fungal growth, 30°C, 48 h; anaerobiosis) and halotolerant LAB on MRS+5% NaCl + 0.01% cycloheximide (30°C, 48 h; anaerobiosis). Enumeration results were subjected to ANOVA analysis and Tukey’s test for mean comparison (*P* < 0.05).

Hygiene and safety quality of the olives was assessed in compliance with EU Regulation n°2073/2005. *Escherichia coli* (TBX, 42°C, 24 h) and coagulase-positive staphylococci (Baird Parker supplemented with Rabbit Plasma Fibrinogen, 37°C, 48 h) were enumerated while *Listeria monocytogenes* and *Salmonella* spp. absence in 25 g was checked for, following ISO 11290-1:2017 and ISO 6579-1:2017 guidelines, respectively.

### Isolation and Identification of Microorganisms

Microorganisms of interest were isolated from three media: halotolerant fungi from YGC+5% salt, halotolerant bacteria from PCA+5% salt and LAB from MRS+5% salt supplemented with 0.01% cycloheximide. For each biological replicate (individual tanks) and media, 20 representative clones (when possible) were isolated from the dilution showing the highest morphological diversity and cryopreserved in 15% (bacteria) or 20% (fungi) (v/v) glycerol at −80°C. A total of 867 yeasts, 16 filamentous fungi and 102 bacteria were collected. A dereplication step was performed for all yeast isolates using Fourier Transform Infra-Red Spectroscopy (FTIR) as described by Coton et al. (2017). FTIR spectrum of each isolate was first compared against an internal dataset of more than 2500 FTIR spectra of reference yeasts to obtain a presumptive identification and secondly against one another to build clusters based on their heterogeneity. Overall, 370 representative fungal isolates (1–5 isolates per cluster) were then chosen and identified by sequencing. Species-level molecular identifications for representative yeast isolates were done after amplification and sequencing of the D1-D2 domain using NL1/NL4 primer pair (Kurtzman and Robnett, 1997) (primer sequences are available in Supplementary Table 1) while all filamentous fungi isolates were identified by targeting the internal transcribed spacer (ITS) region using ITS4/ITS5 primers (White et al., 1990). All bacterial isolates were identified by 16S rRNA gene sequencing after amplification with fD1 and rP2 primers (Weisburg et al., 1991).

Sequences were assembled into contigs using Geneious software^1^ (Kearse et al., 2012) and compared with the GenBank database using the “Basic Local Alignment Search Tool” (BLAST)^2^ and applying a 97% identity and 98% coverage threshold for species identifications. The obtained genus- or species-level identifications of yeasts were then confronted with FTIR dendrogram analyses. When identifications were consistent inside a given cluster, the identification was expanded to all the isolates within the cluster.

### Metabarcoding Analysis on Olive Fruits and Brine During Fermentation

#### Sample Preparation and Total DNA Extraction

Metabarcoding analysis was performed for all sampling points on brine and olive fruits (*n* = 216 samples) separately. Brine samples (5 mL) were centrifuged at 9,000 *g* for 15 min at 4°C and the obtained pellets were washed in TS diluent before storage at −20°C. Regarding olive samples, 10 g of olive surface pulp were collected and ground with 40 mL of TS diluent using an Ultra-turrax homogenizer (T25, Ika Works Inc., United States). Then, the mix was further homogenized for 1 min in a Stomacher Bag® (Interscience, St-Nom, France). Aliquots of 5 mL were then centrifuged (600 *g*, 5 min, 4°C). The supernatants were recovered and centrifuged (9,000 *g*, 15 min, 4°C) to obtain cell pellets which were stored at −20°C until DNA extraction. Total DNA extractions and purifications were performed using NucleoSpin Soil DNA kit (Macherey-Nagel, Germany) with a supplementary initial enzymatic lysis. First, pellets were thawed at room temperature and immediately resuspended in 400 μL of lysis buffer (Tris-HCl 20 mM at pH 8.0, EDTA 2 mM, Triton X-100 1.2%) supplemented with lysozyme (20 mg/mL) and mutanolysin (5 U/μL), then RNase A (25 μg/mL; Qiagen, Germany) and lyticase (0.5 U/μL; Sigma-Aldrich, Germany) were added. Samples were incubated at 37°C for up to 2 h followed by a mechanical lysis step in NucleoSpin® Bead Tubes Type A using a Retsch MM400 mixer mill. Then, proteinase K (20 mg/mL) was added to each sample, followed by incubation for 1 h at 56°C. The rest of the extraction and purification was performed according to the kit manufacturer’s instructions and DNA extracts were stored at −20°C.

#### Amplification and Sequencing Parameters

To study bacterial and fungal diversity, DNA extracts were amplified by PCR using universal primers. For bacteria, the V3-V4 region of the 16S rRNA gene was targeted using S-D-bact-0341-b-S-17 and S-D-BAct-0785-a-A-21 primers and the PCR conditions described by Klindworth et al. (2013). For fungi, ITS3f/ITS4_Kyo1 primers targeting the ITS2 region were used according to the PCR conditions described by Toju et al. (2012). ITS2 and V3-V4 16S rRNA amplicons were sequenced at GATC sequencing platform (Eurofins, Germany) using Illumina Miseq PE300 technology generating 2 × 300 bp reads and a total of 4.8 and 4.4 Gb of data for bacterial and fungal amplicons, respectively.

#### Bioinformatics

Sequences were pre-processed for quality and length using the following parameters: amplicon size between 370 and 490 bp for V3-V4 contigs and 100–530 bp for ITS2 contigs, mismatch rate was set at 0.1 and sequences with NNN were filtered out for both data types. Sequencing data were then analyzed using the FROGS pipelines as developed by Escudié et al. (2018) under Galaxy (Afgan et al., 2018). Briefly, raw paired-end reads were assembled and sequences were clustered using the Swarm algorithm (Mahé et al., 2015) with an aggregation distance of 3 into Operational Taxonomic Units (OTUs). Sequences underwent some filtering steps: chimeras were detected using Uparse (Edgar, 2013) and “de novo parameter” and removed, then sequences with a relative abundance below 5 × 10^–5^ or present in only one sample were excluded. For ITS2 data, ITSx was used prior to affiliation to extract and select ITS2 sequences (Bengtsson-Palme et al., 2013). Finally, affiliation step was performed using SILVA (V138) and UNITE 8.2 fungi databases for 16S and ITS2 data, respectively. When species identification by blastn+ and identification was below 97%, affiliation was manually corrected to the genus. In the same manner, when sequences were multi-affiliated by FROGS, because the targeted V3-V4 region or ITS2 were unable to discriminate species, the resulting species level assignations were implemented into the final OTU table. OTUs affiliated to chloroplasts or mitochondrial sequences in the 16S data set were excluded.

#### Biodiversity and Statistical Analyses

Processing and statistical analyses of microbial communities were performed using Phyloseq package (McMurdie and Holmes, 2013) under R software. For alpha and beta-diversity analyses, data were first normalized based on the sample that had the lowest number of sequences. Taxonomic composition and abundance distribution were then determined and alpha-diversity indexes (Chao1 and Shannon) calculated. Kruskal-Wallis test was used to compare values according to four variables: matrix, harvest period, olive type and fermentation stage. When differences were significant (*P* < 0.05), the Wilcoxon test was performed to explore differences within the variable. To analyze beta-diversity, unweighted Unifrac and weighted Unifrac distances were calculated. Principal Coordinate Analysis (PCoA) and Adonis test (999 permutations) in R vegan package (Oksanen et al., 2013), were then used to assess the impact of the same four variables than for alpha-diversity. Moreover, OTU abundance difference analysis under the same four variables was performed on the non-rarefied OTU count table using Deseq2 package (Love et al., 2014; McMurdie and Holmes, 2014; Safari et al., 2020). Differences were expressed as log2 fold changes and their significance levels were tested using Wald test followed by Benjamini-Hochberg False Discovery Rate correction with alpha value set at 0.05 (Benjamini and Hochberg, 1995). Taxonomic composition visualization for bacterial communities were obtained using Krona hierarchical data browser (Ondov et al., 2011) and ggplot2 package (Wickham, 2016).

### Biochemical Analyses

#### Brine pH Measurements

Brine samples from each sampling point were analyzed for pH using a pH meter (Hanna Instruments HI 2020-02). Three independent measures were done for the four fermentations. The reported values are the average of three replicates per sampling point and condition.

#### Detection and Quantification of Phenolic Compounds in Olive Fruits

Oleuropein, hydroxytyrosol and tyrosol were identified and quantified in olive fruits by HPLC-Diode Array Detector. Extractions were performed from 2 g of olive pulp in DMSO as described by Crawford et al. (2018) except that extracts were not diluted in water before injecting. Analyses were performed on a HPLC Agilent 1100 series (Agilent technologies, Santa Clara, CA, United States) equipped with a Luna C18(2) column (5 μm, 4.6 mm × 150 mm) (Phenomenex Inc., Australia) and a diode-array detector (280 nm). All injection, elution and detection parameters were set as described by Crawford et al. (2018). For quantification, a standard mix of the three phenolic compounds at 1 mg/mL was prepared in methanol and the linear range built by injecting dilutions ranging from 0.1 to 100 μg/mL in duplicate. A 10 μg/mL dilution was used as quality control and injected regularly during analysis. Quantitative results were subjected to ANOVA analysis and Tukey’s test for mean comparison (*P* < 0.05).

#### Organic Acid Quantification in Brine During Fermentation

##### Sample preparation

Eight organic acids were quantified in brines throughout fermentation. For each sample, 2 mL of brine were filtered on 0.45-μm cellulose acetate membrane and subjected to LC-MS and HPLC analyses by direct injection in the columns.

##### LC-MS analyses

Detection and quantification of four organic acids (gluconic, glucuronic, malic and succinic acids) were performed on a 1260 Infinity binary HPLC and a 6530 Accurate Mass LC-QToF-MS (Agilent Technologies). Compound separation was performed on a Rezex ROA-Organic acid column (150 mm × 4.6 mm) (Phenomenex Inc., Australia) and the mass spectrometer operated in negative electrospray ionization mode. Analyses were performed under the following conditions: injection volume 5 μL, flow at 0.3 mL/min, isocratic elution in H_2_O + 0.1 % formic acid and 15 min run time. Identification and quantification were achieved using standard solutions of the four organic acids mixed at concentrations ranging from 0 to 1 mg/mL. To determine the matrix effect, standards solutions were prepared in H_2_O + 0.1 % formic acid and in H_2_O + 5% NaCl to mimic brine and injected in triplicates for each concentration. As salt presence had a negative impact on the analyte response, only standard mixes prepared in salt (matrix matched calibration curve) were kept to build the linear range used for quantification. Furthermore, to ensure quantification, a standard mix at 20 μg/mL was used as a quality control sample and injected regularly during the analyses. For each compound, the limits of detection (LOD) and quantification (LOQ) were determined based on the standard deviation of the analyte response and the standard deviation slope. Quantitative results were subjected to ANOVA analysis and Tukey’s test for mean comparison (*P* < 0.05).

##### HPLC analyses

Citric, oxalic, acetic and lactic acids were detected and quantified using an HPLC-RID Agilent 1200 system (Agilent technologies, Santa Clara, CA, United States). Briefly, 5 μL of filtered brine were injected in a Rezex ROA-Organic acid column (150 mm × 4.6 mm) (Phenomenex Inc., Australia). Mobile phase was 0.01M H_2_SO_4_ at a flow rate of 0.6 mL/min and the run time was 25 min. Signals were recorded using a refractive index detector. Standards solutions of the four acids, first individually and then mixed at six different concentrations, were injected in duplicate in order to build a linear range. Quantification was based on the injection of an external standard mix of the four molecules at 1 mg/mL regularly during analysis. Quantitative results were subjected to ANOVA analysis and Tukey’s test for mean comparison (*P* < 0.05).

#### Volatile Compound Profile Analyses in Olive Fruit

Volatile profiles of olive fruits were obtained using Headspace GC-MS. Compound extraction was performed using a Perkin Elmer Turbomatrix HS-40 trap automatic headspace sampler with trap enrichment on 2.5 g of olive fruits placed in 22 mL vials. Analyses were performed according to methods previously described by Harlé et al. (2020). Prior to compound identification, data were processed using PerkinElmer Turbomass software, version 5.4.2.1617 and by converting the raw data to time- and mass-aligned chromatographic peak areas using the open-source XCMS package implemented with the R statistical language (Smith et al., 2006). Parameters were set as follows: full width at half maximum = 5, group bandwidth = 3, span = 0.2, signal to noise ratio = 5. Volatile compound identification was achieved by comparing the retention index and mass spectral values (1) from the NIST 2008 Mass Spectral Library (Scientific Instrument Services, Ringoes, NJ, United States) with a threshold set at 65 % and (2) when possible with those of authentic standards (Sigma Aldrich, France) analyzed in the same system and those reported in the literature. Volatile profile changes between samples were investigated by comparing abundance of the identified compounds using PCA and ANOVA analyses under R software. Hierarchical clustering and correlation analyses of the compounds showing a significant difference throughout fermentation was performed using Ward’s minimum variance linkage and Euclidean distance method under R software.

### Correlation Analyses Between Microbial and Biochemical Data

Correlation analyses were performed separately for brine and olive data. First, principal component analysis (PCA) was implemented to assess overall differences between samples using both culture-independent microbial data and biochemical data. Then, Spearman rank correlation ρ coefficients between microbial species (relative abundance obtained by culture-independent approach) and biochemical compounds were calculated. *P* values were adjusted using Benjamini-Hochberg correction and significance level was set at 0.05 (Benjamini and Hochberg, 1995). Results for species showing at least one significant correlation were visualized on a heat-map with hierarchical clustering using Ward’s minimum variance linkage and Euclidean distance method. Correlations with *P* > 0.05 were considered null and set to ρ = 0. All analyses were performed with R software using FactoMiner, Factoextra, Hmisc, Psych and gplots packages (Lê et al., 2008; Wickham, 2016).

## Results

### Microbial Dynamics During Fermentation Through Culture-Dependent Approach

**FIGURE 1:**
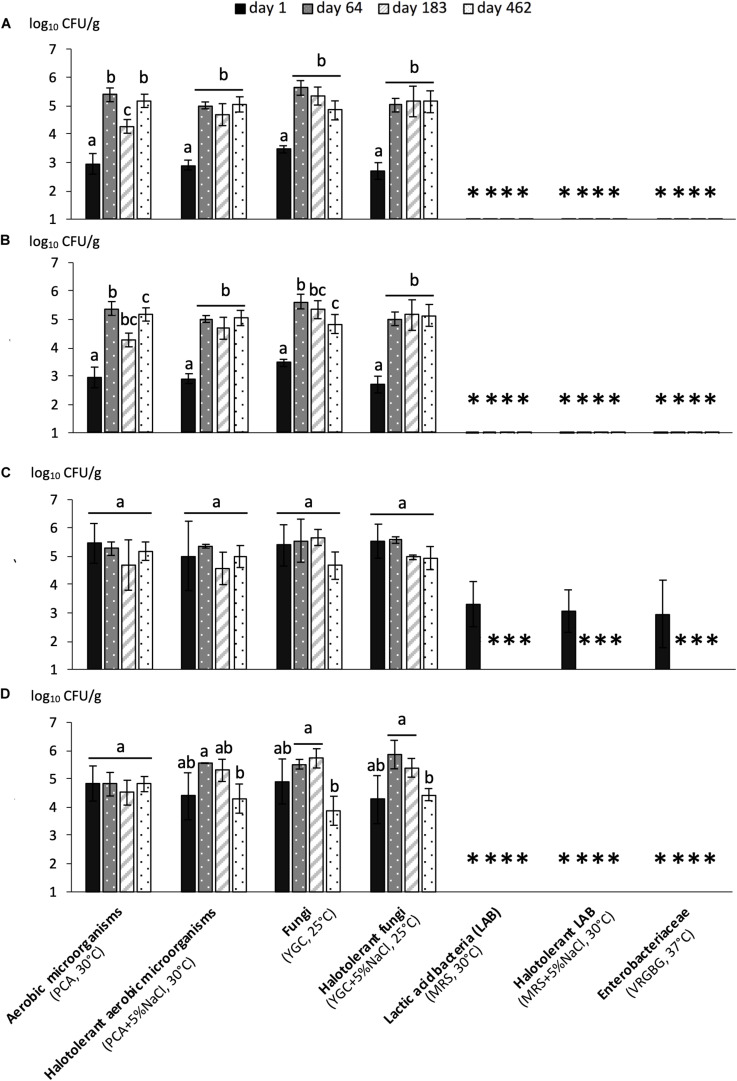
Microbial counts (mean values of 3 replicates ± standard deviation) during fermentation of organically or conventionally grown Nyons olives harvested at early or late stages. **(A)** R1-Orga: Early harvest-Organic olives, **(B)** R1-Conv: Early harvest-Conventional olives, **(C)** R2-Orga: Late harvest-Organic olives and **(D)** R2-Conv: Late harvest-Conventional olives. Values are expressed as CFU per g of mixed brine and olive (1:1 w/w ratio). For each fermentation, different letters for a given population indicate significantly different counts (*P* < 0.05) under Tukey’s HSD test. “^∗^” symbol indicates counts under the detection threshold.

Microbial populations were monitored for the four fermentations – early harvest with organic olives (R1-Orga), early harvest with conventional olives (R1-Conv), late harvest with organic olives (R2-Orga) and late harvest with conventional olives (R2-Conv). Overall, fungal populations were found at the highest concentrations (nearly 5 log_10_ CFU/g mixed olives and brine) and represented the main population during all fermentations (Figure 1). While no major difference in counts was observed between conventional and organic olives (*P* > 0.05), some differences were observed between harvests. Initial microbial loads on fresh fruits were very different between early and late harvests, with up to ∼3 log_10_ lower counts for fungal and total mesophilic and halotolerant populations for the early harvest (*P* < 0.05). However, similar fungal and bacterial populations were reached by 64 days (around 5.6 log_10_ CFU/g) and remained fairly stable, especially for early harvest fermentations, while a slight decrease in populations was observed (1–2 log_10_) for the late harvest (R2 samples) between day 183 and 482 (*P* < 0.05). One other major difference was the presence, although at low concentrations, of LAB (3 log_10_ CFU/g) and enterobacteria (2.9 log_10_ CFU/g) on R2-Orga fresh fruits, while undetected in other samples. However, both LAB and enterobacteria populations rapidly decreased to values below the detection limit (2.3 and 1 log_10_ CFU/g, respectively) by day 64 and remained undetected for the rest of the fermentations regardless of harvest period or olive type. Overall, no major difference was observed between halotolerant and total microbial populations.

### Fungal Community Diversity and Dynamics During Fermentations

#### Fungal Biodiversity Determined by Fungal Isolate Identifications

**FIGURE 2:**
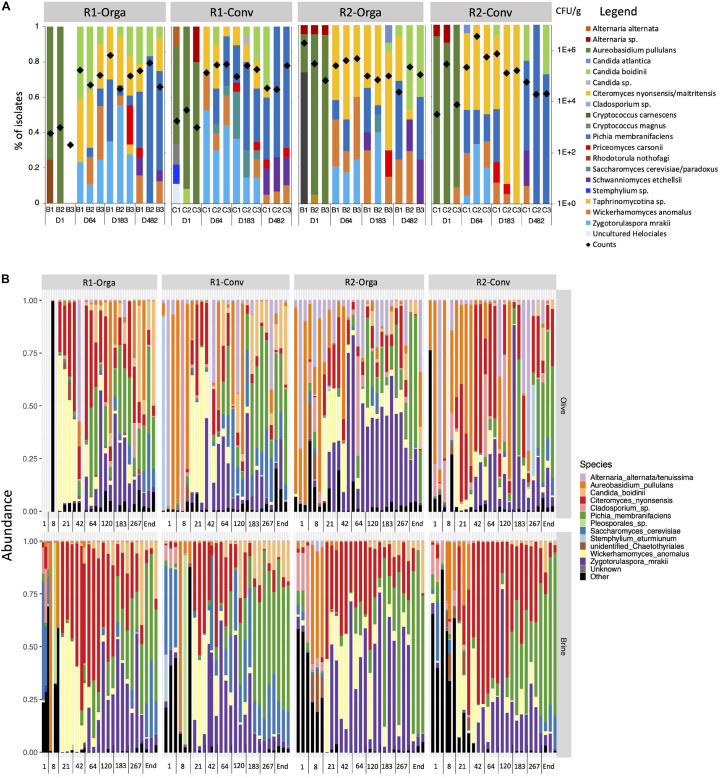
Fungal diversity and dynamics during Nyons table olive fermentations revealed by **(A)** culture-dependent approach and **(B)** ITS2 metabarcoding analysis. Results are presented for the four studied fermentation productions: R1, early harvest; R2, late harvest; Orga, organically grown olives; Conv, conventionally grown olives. Diamond symbols indicate fungal population counts in log CFU/g.

A total of 867 fungal isolates were cryopreserved, dereplicated and identified to assess fungal diversity and composition for each fermentation condition (Figure 2A). Similar trends were observed for all fermentations. Filamentous fungi dominated at the start of the fermentation and *Aureobasidium pullulans* was highly abundant and ranged from 20 to 100% in the monitored tanks. Minor species were also encountered such as *Cryptococcus magnus*, *Cryptococcus carnescens* and *Alternaria* sp. in 7 out of the 12 fermentation tanks. However, from day 64 onward, all of the species mentioned above were no longer detected and the fermentations were dominated by four yeast species, *Wickerhamomyces anomalus*, *Pichia membranifaciens*, *C. nyonsensis* and *Zygotorulaspora mrakii*, although individual abundances varied between replicates and according to olive types and harvest periods. For instance, *C. nyonsensis* abundance was particularly high in the late harvest R2 tanks representing, at day 64, 50% of fungi, before reaching 100% at day 183 for the R2-Conv fermentation. However, fungal dynamics shifted towards the end of fermentation. *Z. mrakii* was no longer detected in any fermentation while *C. nyonsensis* and *W. anomalus* abundances decreased. In the end, *P. membranifaciens* remained the dominant species. *Schwanniomyces etchellsii* and *Candida boidinii*, which were subdominant in the first fermentation stages, also increased and represented up to 40% abundance in the case of *C. boidinii.* Other species such as *Saccharomyces cerevisiae/paradoxus* and *Priceomyces carsonii*, were identified in multiple tanks, although they were not specific to one fermentation condition (i.e., olive type) or stage.

#### Fungal Dynamics Using ITS2 Metabarcoding Analysis

A total of 6 010 233 quality-filtered contigs were obtained through ITS2 Illumina sequencing After chimera and singleton removal, sequences were clustered into 134 OTUs belonging to Ascomycota (97%), Basidiomycota (2.5%) while 0.5% were unidentified (Supplementary Figure 1). After normalization, 1302 sequences per sample were kept for diversity analyses.

Alpha-diversity indexes (Chao1 for richness, Shannon for evenness) did not show any significant differences according to the harvest period or olive type and only a slight difference between olive fruits and brines (Supplementary Figure 2). However, Shannon index was significantly different according to fermentation stage (*P* < 0.05) and when both matrix and fermentation stage were considered (*P* < 0.001) for the two indexes. Thus, at day 1, Shannon index was higher in brines compared to olive fruits. Both Chao1 richness and Shannon evenness indexes significantly decreased from day 1 to day 21 in brines and olive fruits (*P* < 0.01). While richness remained stable until the end of fermentation, evenness in brine and olive samples significantly increased from day 42 until day 183.

These observations were further confirmed by beta-diversity analyses performed on unweighted Unifrac and weighted Unifrac distances. All four studied variables (i.e., matrix, harvest period, olive type and stage) were significantly different according to the Adonis test (*P* < 0.05). However, for harvest period, type and matrix, the associated *R*^2^ values, which reflect the percentage of variance between distances explained by the tested variable, were very low (*R*^2^ < 0.05), hence implicating a weak difference overall. The impact of fermentation stage was, however, higher (*R*^2^ > 0.5 for weighted Unifrac) and could be visualized on PCoA plots (Supplementary Figure 3). A gradient following fermentation process could be seen along axis 1 for both unweighted Unifrac (19.8%) and weighted Unifrac (47.5%) plots, separating day 1 and day 8 samples from the other fermentation stages. Samples from day 21 to the end of fermentation were better separated by weighted Unifrac (axis 28.7%) compared to unweighted Unifrac distances. The major difference between the observed distances is due to the OTU relative abundances and composition. This implies that differences in fungal communities are rather qualitative in the early days of fermentation while fungal community profiles differed by OTU abundances during the later stages of fermentation. Further analyses were therefore done to investigate fungal community composition differences according to fermentation stage and matrix interactions (Figure 2B). Overall, the fungal community profile strongly differed between olive fruit samples and brines during the first days of fermentation (days 1 and 8). High genus diversity was observed in olive fruits and they mainly corresponded to plant-associated filamentous fungi (i.e., *Epicoccum*, *Cladosporium*, *Leucosporidium, Penicillium* and *Filobasidium*) although *Aureobasidium pullulans* dominated in both matrices. From day 42 to the end of the fermentation, *Z. mrakii*, *W. anomalus* and *P. membranifiaciens* abundances were significantly higher in brines compared to olive fruits (*P* < 0.001), but the same shifts in species composition were observed in both matrices. For example, between days 8 and 21, *W. anomalus* and *C. nyonsensis* considerably increased and dominated the fermentation (log2 fold change |13.3| and |10.6| in brines, respectively, *P* < 0.001). However, *W. anomalus* progressively decreased from day 42 onward, while *C. nyonsensis* abundances decreased from day 64 to day 120. On the other hand, *Z. mrakii* increased in both olive and brine samples from day 42 and dominated until day 183. From day 183 to the end of the fermentation, *P*. *membranifaciens* dominated in both matrices and represented up to nearly 50 and 70% of the relative abundance in olives and brines, respectively. Noteworthy, early and late harvests significantly differed in abundances of two subdominant species. Indeed, *S. cerevisiae* (log2 fold change |3.9|, *P* < 0.001) and *C. boidinii* (log2 fold change |1.6|, *P* < 0.001), which abundances increased toward the end of fermentation, were higher in R2 than in R1 harvests.

### Bacterial Community Succession in Olives and Brines During Fermentations

#### Bacterial Dynamics Using Culture-Dependent Analyses

**FIGURE 3:**
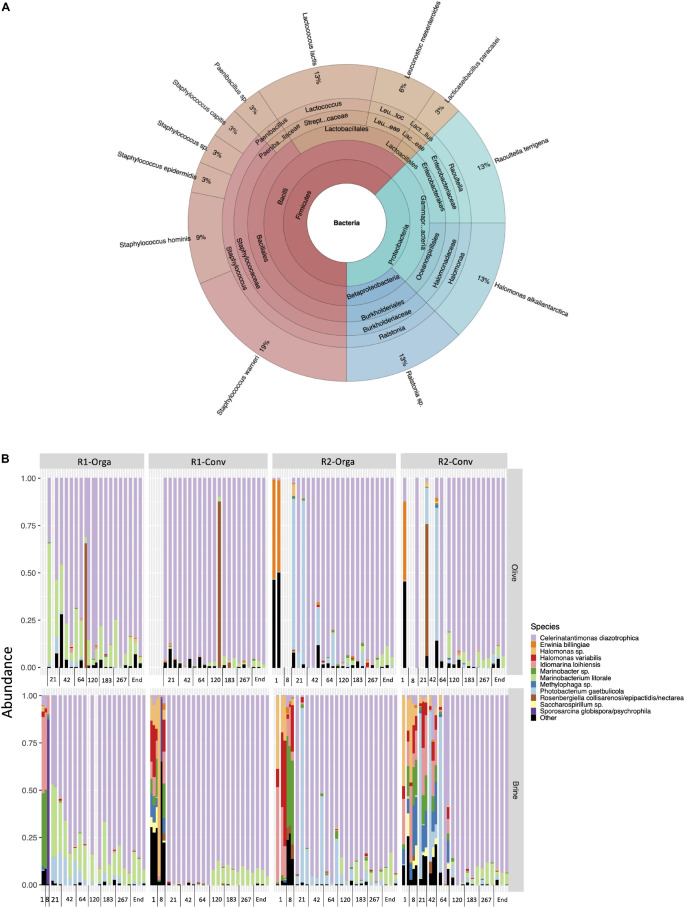
Nyons table olive bacterial community composition: **(A)** Global composition based on culture-dependent analysis and isolate identification from PCA+5% NaCl. **(B)** Top 12 bacterial species during the four studied fermentation productions: R1, early harvest; R2, late harvest; Orga, organically grown olives; Conv, conventionally grown olives.

As previously stated, bacterial populations were low compared to fungal populations and only a limited number of bacteria were isolated, mainly from the early stages of fermentation (days 1 and 64). Coagulase-negative staphylococci were the most represented genus (*Staphylococcus warneri*, *Staphylococcus epidermidis*, *Staphylococcus hominis*), although all isolates were identified at day 64 (Figure 3A). Soil bacteria (*Ralstonia* and *Raoultella* genera) represented 26% of bacterial isolates while marine bacteria represented 13% (*Halomonas alkaliantartica*). Some LAB species, namely *Lactococcus lactis*, *Leuconostoc mesenteroides* and *Lacticaseibacillus paracasei*, were also identified (21% of all isolates) but only from organic fresh olive fruits and brine samples (Supplementary Table 2). Hygiene and safety criteria, i.e., coagulase-positive staphylococci levels, absence of pathogens, were satisfied at all sampling times considered including in the final product (data not shown).

#### Bacterial Dynamics Using Metabarcoding Analysis

V3-V4 16S rDNA gene Illumina sequencing resulted in 7 941 784 reads which passed quality filters with an average length of 427 bp. After assignation and contaminant removal, 2 828 839 sequences were left and clustered into 54 OTUs. Proteobacteria were the most represented phylum with 99.75 % of sequences, the remaining 0.25% belonging to Firmicutes. Among Proteobacteria, OTUs related to *Celerinatantimonas diazotrophica* were dominant (78% of sequences), followed by *Photobacterium gaetbulicola* (7%) and *Marinobacterium litorale.* Firmicutes mainly consisted of *Bacillus* sp. and LAB with *L. mesenteroides* (9%), *L. lactis* (9%) and *Lactiplantibacillus pentosus* (8%) (Supplementary Figure 4).

Sequence numbers per sample were very heterogeneous, especially for olive samples, thus samples with less than 200 sequences were excluded (*n* = 32). All remaining samples were rarefied to 207 sequences per sample (corresponding to the sample with the lowest sequence number) to conduct alpha and beta-diversity analyses. Neither matrix nor harvest had a significant impact on alpha-diversity or beta-diversity (*R*^2^ < 0.01) of bacterial communities. As for olive type, richness and evenness were significantly higher in the organic olive fermentations compared to conventional ones (*P* < 0.01). *P. gaetbulicola* was found in higher abundances in organic olive fermentations when compared to conventional ones (log2 fold change |4|; *P* < 0.05). Noteworthy, R1-Conv fermentation showing the lowest alpha-diversity indexes and differed significantly according to the fermentation stage. Fermentation stage was the variable with the most impact on bacterial communities. Based on weighted Unifrac distance, it explained most of the observed variance (*R*^2^ = 0.26). Both richness and evenness were highest during the first stages of fermentation (days 1 and 8) and this was further confirmed by beta-diversity analysis. PCoA plot based on weighted Unifrac matrix showed a clear opposition on axis 1 (54.3%) of brine at days 1 and 8 and all other fermentation stages (Supplementary Figure 5). The bacterial community composition plot (Figure 3B) revealed that these samples had a completely different profile. They were mainly composed by *Halomonas*, *Iodomarina*, *Marinobacter* and *Methylophaga* genera. Following day 8, alpha-diversity significantly decreased (*P* < 0.01) and stabilized around 0.25 for Shannon index. All the previously mentioned species decreased in the following stages except for the late harvest conventional (R2-Conv) fermentation where they were identified until day 42. Overall, the composition of the bacterial communities was similar in all cases from day 21 to the end of fermentation. *C. diazotrophica* clearly dominated (between 45 and 99% abundances) while *M. litorale* abundances remained stable.

### Phenolic Compounds Changes During Fermentation

**FIGURE 4:**
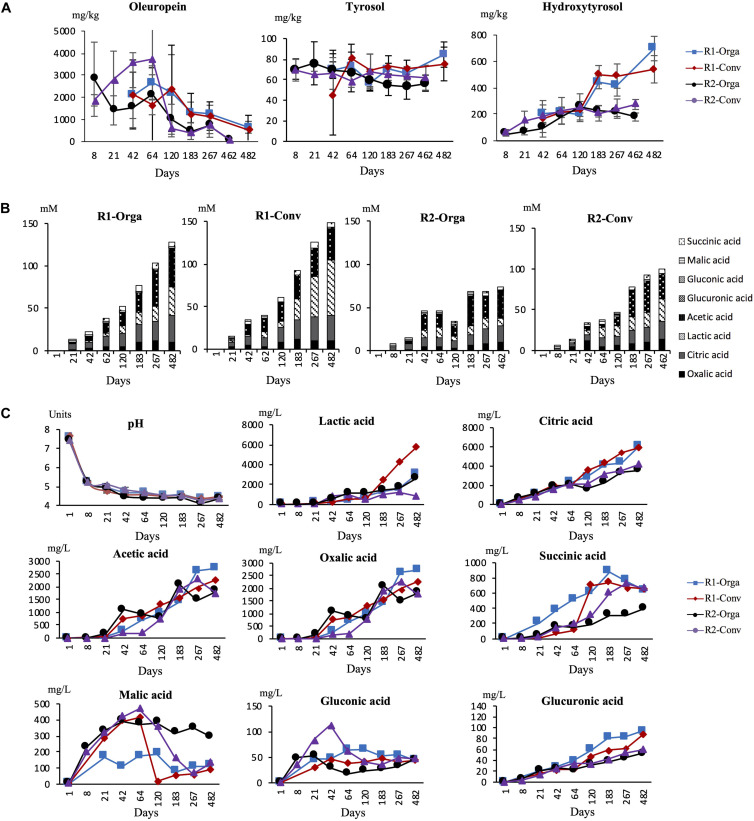
pH and biochemical concentration changes during Nyons table olive fermentations. **(A)** Phenolic compound concentration in olive fruit during fermentation in mg/kg of olive pulp, **(B)** Total acid concentration in brine in mM and **(C)** pH/individual acid concentration evolution in mg/L of brine. R1-Orga, early harvest-organic olives; R1-Conv, early harvest–conventional olives; R2-Orga, late harvest-organic olives; R2-Orga, late harvest conventional olives. Data are expressed as mean values from triplicate (tanks) determinations.

Changes in oleuropein concentrations, and its derivative compounds, tyrosol and hydroxytyrosol, were quantified at each sampling date and results are shown in Figure 4A. Overall, similar trends were observed for all three molecules regardless of the fermentation conditions. Oleuropein was found at high concentrations ranging from 1 to 4 g/kg of olives during the first months of fermentation, although at highly variable concentrations among the four fermentations. From day 120 to the end of the process, concentrations decreased progressively for both harvests, although this was only significant for the late harvest. As a consequence, concentrations significantly differed (*P* < 0.05) at the final fermentation day. For the early harvest R1, from day 120 to 462, the concentration was divided by 5 (around 500 mg/kg olive). In comparison, the observed decrease started at day 64 for the late harvest R2 and the amount was 15–20 times lower, around 100 mg/kg olive, by the end of the fermentation. Inversely, the hydroxytyrosol concentration progressively increased, even though, once again, differences were pronounced between harvests. For early harvest R1, concentrations were significantly higher from day 183 onward and multiplied by 5 by the end of the fermentation (around 600 mg/kg) whereas for late harvest R2, the concentration increased 2-fold between days 8 and 120 then was relatively stable until the end of the fermentation (around 200 mg/kg). Tyrosol concentration remained fairly stable (*P* > 0.05) and was quantified at a lower concentration than the two other compounds, between 50 and 70 mg/kg olive. No significant differences were observed between either harvests or olive types.

### pH Evolution and Organic Acids Changes in Brine During Fermentation

For all fermentation tanks, pH was measured at regular time intervals. Initial brine pH was 7.7 units, then dropped during the first week of fermentation to 5.2 units. From day 8, pH declined slowly oscillating between 4.2 and 4.4 until the end of the fermentation regardless of the harvest or olive type.

In addition, eight organic acids known to be relevant in olive fermentations were monitored and quantified at different times in brine samples. Total acids produced during fermentation were calculated and are presented in Figure 4B. As expected, none of the acids were detected in brines at the start of fermentation (day 1). After day 8, total acids increased continuously to reach ∼127, 148 and ∼100 mM by the end of the fermentation for R1-Orga, R1-Conv and R2-Orga fermentations, respectively. However, the late harvest R2-Orga fermentation showed a different trend as, after a slight increase of total acids up to day 64 (40 mM), the concentration temporarily decreased before reaching 62 mM at the end of fermentation.

When considering individual acids, all of them significantly increased during fermentation (Figure 4C). Significant differences were observed for five out of eight acids between harvest periods R1 and R2 (*P* < 0.01), while significant differences were only observed for lactic and succinic acids between conventional and organic olive fermentations (*P* < 0.05) (Supplementary Table 3). Citric, acetic and lactic acids showed the highest concentrations, over 1 g/L of brine, during most of the fermentations. However, their concentrations were higher for the early harvest R1 samples. Indeed, citric acid was detected as early as 8 days in brine and continuously increased to reach ∼6 g/L at the end of this fermentation versus ∼3 g/L for late harvest R2 fermentations. Acetic acid was detected after 21 days in all fermentations except for the late harvest R2-Conv fermentation (detected at 42 days) and gradually increased to ∼2 g/L by 267 days. Lactic acid concentrations differed between harvests and olive types. While this acid was detected by day 8 in early harvest R1 fermentations, it was only produced after 42 days for the late harvest R2 fermentations. Moreover, the levels reached were 3 to 6-fold significantly lower, especially for organic olives, by the end of fermentation. Succinic and oxalic acids were detected in the early stages of fermentation and concentrations ranged between 0.1 and 1 g/L during fermentation. For both acids, concentrations continuously increased during fermentation although it was only significantly different from earlier sampling times at around 120 days, except for the late harvest R2-Conv fermentation. Gluconic and glucuronic acids were detected at lower concentrations compared to other acids. Moreover, they did not exceed 30 mg/L brine and remained stable after an initial increase during the first weeks of fermentation. In a similar manner, malic acid, despite higher levels in the early stages of fermentation (0.4 g/L), did not significantly evolve over time except for the early harvest R1-Conv fermentation where the concentration dropped between days 64 and 120.

### Changes in Volatile Profiles During Nyons Olive Fermentations

A total of 67 compounds were detected during fermentation belonging to different families: 27 esters, 11 aldehydes, 8 short fatty acids, 6 alcohols, 4 ketones, 4 phenols and 6 to other classes. Sample projection using Principal Component Analysis showed that the first dimension (40.8%) clearly separated the different sampling times, from day 1 on the left to the final day of fermentation on the right (Supplementary Figure 6A). In PCA plots and considering dimensions 1 and 3 (8.4%), samples belonging to harvest R1 were separated from harvest R2 (Supplementary Figure 6B). Results thus suggest that, in addition to fermentation stage, harvest period also impacted the volatile profile. Moreover, abundance significantly differed for 26 and 22 compounds between harvest periods and olive types, respectively, while 62 were impacted by fermentation stage (Supplementary Table 4). However, for compounds with significantly different abundances between harvest periods, the trends were similar throughout the fermentation.

**FIGURE 5:**
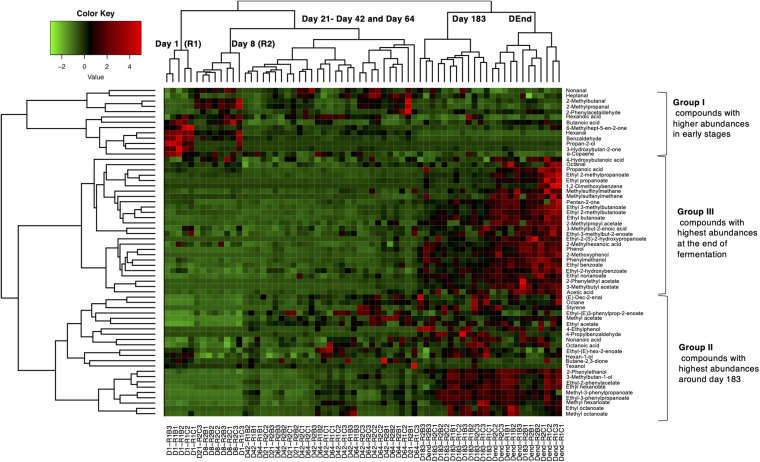
Normalized heat-map representation of volatile abundance changes during Nyons table olive fermentations. Hierarchical clustering was performed using Ward’s Linkage and Euclidean distance. Sample names are at the bottom whereas compounds are on the right part of the map. Color ranging from red to green indicates low to high abundances.

To further investigate volatile compound dynamics, hierarchical clustering analysis with a heatmap representation was performed on the 62 compounds presenting significant differences in abundances during fermentation (Figure 5). Three main volatile clusters were distinguished and correlated with the different fermentation stages. Group I gathered 13 molecules, mainly aldehydes such as hexanal and benzaldehyde. Their abundances were higher during the early stages (day 1 and day 8) of fermentation and decreased afterward. Group II was composed of 23 compounds mostly belonging to esters (*n* = 10) and alcohols (*n* = 4) and their abundances increased during intermediate fermentation stages (from day 21 to 64) then remained high until the end of the fermentation. Finally, Group III was composed of 26 compounds mainly esters (*n* = 13) and short-chain fatty acids (*n* = 5) for which their respective abundances were the highest during the final stages of the fermentation.

### Correlation Between Biochemical Profile and Microbiota During the Fermentation

A principal component analysis was performed with all microbial and biochemical data to correlate temporal changes in microbial communities with biochemical profiles of brine (Supplementary Figure 7A) and olive fruits (Supplementary Figure 7B). Concerning brine, dimensions 1 and 4, which explained 18.9 and 6 % of the variance respectively, provided the best separation of samples according to fermentation stages. A clear opposition was seen between early stages of fermentation (day 1 and day 8) and final stages of fermentation (days 183 to end of fermentation). While early stages of fermentation were associated with most of the plant-associated species and pH, four species, namely *C. diazotrophica*, *P. membranifaciens*, *C. boidinii* and *P. carsonii*, stood out in the final stages and were closely related to the production of most acids. Noteworthy, *C. nyonsensis* and *W. anomalus*, between days 21 and 42, were not related to any biochemical variable. Concerning olive fruit biochemical and microbial profiles, similar clustering was observed along dimensions 1 (28%) and 2 (10.1%) and the same four species stood out in regards to the formation of 14 esters, two ketones, two phenyl alcohols and one phenolic compound hydroxytyrosol. Interestingly, five compounds were closely related to axis 2 including oleuropein, however, without any link to a given species.

**FIGURE 6:**
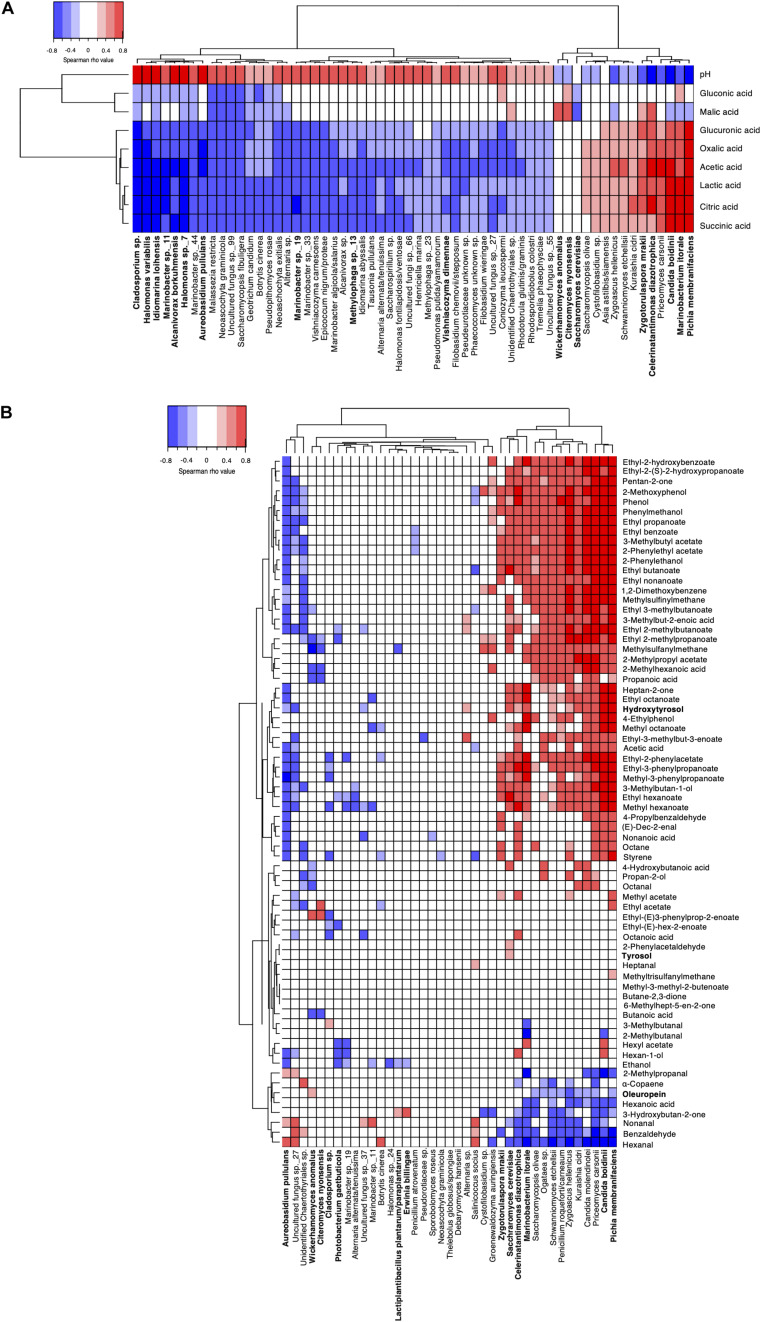
Spearman correlation matrices between microbial species composition and biochemical compounds in panel **(A)** brine and in panel **(B)** olive fruit. Only significant correlations are shown (FDR-corrected *P* < 0.05). Species with relative abundances >0.5% are in bold. Positive correlations are indicated in red while negative ones are indicated in blue. In panel **(B)**, phenolic compounds quantified by HPLC are indicated in bold while volatiles quantified by GC-MS are in plain characters.

To get better insight into the potential links between aroma compounds and microorganisms, Spearman’s correlations were also calculated between microbial species and organic acids, pH in brine (Figure 6A), and between phenolic and volatile compounds in olive fruits (Figure 6B). Regarding changes in brines, significant negative correlations were found between organic acids and 45 fungal and bacterial species. The strongest antagonisms (|ρ| > 0.6) were observed for *Cladosporium* sp. and *A. pullulans* for fungi and *Halomonas* spp., *Iodiomarina loihensis*, and *Alcanivorax borkuhmensis* for bacteria. Overall, eleven species showed at least one positive correlation with the acids. The strongest correlations were observed for *P. membranifaciens* with ρ values above 0.6 for citric, succinic, gluconic, oxalic acids and above 0.7 for acetic and lactic acids. The yeast *C. boidinii* and the bacterium *M. litorale* showed similar correlation levels for lactic, citric and succinic acids, while *Z. mrakii* and *C. diazotrophica* were well correlated with acetic, citric and lactic acids (0.4 < |ρ| < 0.6). *W. anomalus* and *C. nyonsensis* were only positively correlated with malic acid (|ρ| ∼ 0.45), whereas no significant positive correlation was observed for *S. cerevisiae.* Strong positive correlations were also sporadically noticed between acids and sub-dominant species. For instance, *P. carsonii* showed a similar correlation profile as *C. boidinii*, while *S. etchellsii* and *Zygoascus hellenicus* were particularly well correlated to acetic acid.

Regarding phenolic compound changes in olive fruits, correlations were limited. Six species, namely *P. membranifaciens*, *Candida molendinolei*, *P. carsonii*, *S. etchellsii*, *Kuraishia cidri* and *Z. hellenicus*, showed a significant negative correlation with oleuropein (|ρ| < 0.4; *P* < 0.05) and positive correlation with hydroxytyrosol (|ρ| > 0.4; *P* < 0.01). *C. diazotrophica*, *S. cerevisiae* and *C. boidinii* were positively correlated with the increase of hydroxytyrosol (|ρ| > 0.6). No significant correlation with tyrosol was determined.

Regarding volatile compounds, three correlation profiles could be distinguished. Some profiles, as observed for *A. pullulans*, associated positive correlations mainly with aldehydes (i.e., nonanal, benzaldehyde) and negative correlations with most volatiles especially esters. An opposite profile was observed for 14 species, i.e., strong negative correlations with aldehydes and strong positive correlations with esters, alcohols and phenolic compounds. Focusing on species with relative abundance > 0.5% and strongest positive correlations (|ρ| > 0.6), highest correlation numbers were observed for *P. membranifaciens* (*n* = 30) and *C. boidinii* (*n* = 27). Correlation levels for both species were particularly high (|ρ| > 0.7) with ethyl octanoate, ethyl benzoate, 2-methoxyphenol, ethyl 3-phenylpropanoate, 2-phenylethanol and phenol. However, *P. membranifaciens* showed further strong correlations with specific esters (ethyl propanoate, 3-methylacetate, ethyl nonanoate, 2-phenylethylacetate, methyl-3-phenylproapanoate), heptan-2-one, pentan-2one and 3-methylbutan-1-ol. By comparison, *C. boidinii* showed the highest correlations with ethyl 2-phenylacetate and ethyl 2-hydroxybenzoate, all species considered. Two yeasts, *Z. mrakii* and *S. cerevisiae*, and two bacteria, *C. diazotrophica* and *M. litorale*, showed several significant correlations although levels were weaker (0.4 < |ρ| < 0.65). *C. diazotrophica* was highly correlated with 2-methoxyphenol, phenol and best correlated with methyl hexanoate and acetic acid compared to other species. *M. litorale* was well correlated with four esters (methyl octanoate, ethyl octanoate, ethyl-2-phenylactete and ethyl 3-phenylpropanoate) and heptan-2-one. As previously observed in brines, subdominant species such as *Z. hellenicus* and *C. molendinolei* showed high correlations (|ρ| > 0.6) with eight and 11 compounds, respectively. In addition, *P. carsonii* showed a correlation profile similar to *C. boidinii* and was the species best correlated with ethyl(2*S*)-2-hydroxypropanoate, 1,2-dimethyoxybenzene, ethyl 2-hydroxybenzoate and phenylmethanol. Noteworthy, for 22 species, including *C. nyonsensis* and *W. anomalus*, only a few or weak correlations were found. Furthermore, 15 volatile compounds showed no significant positive correlation (*P* > 0.05) with any of the species.

## Discussion

Empirically, the olive fermentation process serves three purposes: reduce natural olive fruit bitterness, preserve it from spoilage by brining and, in doing so, enhance the organoleptic properties of the final product and hence make it edible. In this work, four complete productions of spontaneously fermented Nyons table olives were studied. The fermentation process in brine was monitored during 15 months for microbial and physicochemical changes to evaluate the impact of autochthonous microorganisms on product characteristics and determine their potential roles as fermentation drivers. The studied fermentations took into account two harvest periods and olive types: organic or conventional. At the end of fermentation, all table olive batches were tasted by the producer and satisfied PDO requirements.

In naturally brined black olives, debittering is essential as no lye treatment is applied. Oleuropein is the main compound responsible for the bitter taste of olive fruits. Its removal during fermentation can be achieved by three phenomena: osmotic diffusion from the olive pulp to the brine, chemical degradation and enzymatic hydrolysis into by-products (Ozdemir et al., 2014). Previous research showed that oleuropein is degraded into non-bitter compounds via either endogenous olive esterase and beta-glucosidase activities (Ramírez et al., 2016) or from microbial enzymatic activities (Johnson and Mitchell, 2018). Among the non-bitter compounds generated, hydroxytyrosol is considered as the main marker of oleuropein degradation (Restuccia et al., 2011) while tyrosol is considered to be among the prevalent phenols in fermented olives. In our study, the monitoring of these compounds directly in olive fruits throughout fermentation revealed that Nyons table olives undergo a slow debittering process. Despite being harvested at full maturity, oleuropein levels were high in fresh fruits and only slowly decreased over time, mostly during the later stages of fermentation. In parallel, hydroxytyrosol concentration increased, confirming degradation of the bitter compound. However, hydroxytyrosol and tyrosol concentrations were relatively low compared to oleuropein decrease and no strong correlations were revealed between these phenolic compound changes and the dominant species (e.g., *P. membranifaciens*, *Z. mrakii*) in the later stages of fermentation. No correlation was neither found for *L. plantarum* and *W. anomalus* although both species were previously reported to possess active hydrolases during black olive fermentation (Bleve et al., 2014; Bonatsou et al., 2015). Based on our findings, debittering during Nyons olive fermentations is most likely due to diffusion and to endogenous enzymes.

Clear shifts in bacterial and fungal communities were observed following brining. Indeed, pH decrease was very fast (within the first weeks) and stabilized at a value below 4.5 units which satisfied PDO requirements. In the same period, olive-plant associated filamentous fungi and *Enterobacteriaceae*, which are two microbial groups found on fresh fruits and considered as common olive microbial spoilers (Medina-Pradas et al., 2017), disappeared. pH decrease was also concomitant with a rapid increase of acid concentrations in brines, especially citric and malic acids, as previously reported by Nychas et al. (2002). Interestingly, both acids were strongly and positively correlated with the dominant yeast species, *W. anomalus* and *C. nyonsensis*, and bacterial species, *C. diazotrophica*, when pH drops in brine. *C. nyonsensis* was first isolated from Nyons table olives (Casaregola et al., 2013) and since then has been identified in other olive fermentations (Arroyo-López et al., 2016) while *W. anomalus* is frequently identified in olive fermentations (Coton et al., 2006; Heperkan, 2013). OTUs related to *C. diazotrophica* dominated the fermentation early on, from 21 days, and remained among the most abundant bacterial species to the end of fermentation. It was first isolated from a high salt environment (Cramer et al., 2011) and, in this study, most likely came from the salt used for the brine. Noteworthy, this bacterium was previously identified in brines of spontaneous olive fermentations (Medina et al., 2016; Anagnostopoulos et al., 2020) with an increasing abundance throughout fermentation but was never the dominant species. However, little is known regarding its role or metabolism in olive fermentations. This species can assimilate glucose, fructose and galactose, which are the main sugars in olives, as well as malic acid and citric acid and also has a beta-glucosidase activity which makes it compatible with Nyons olive fermentation conditions. In our case, strongest significant positive correlations were determined with citric, acetic and lactic acids in brine. The correlation with lactic acid is particularly interesting. Two other microorganisms, namely *P. membranifaciens* and *C. boidinii* were closely correlated to these compounds. However, if citric acid and acetic acid production by these two species is known, they are not reported to produce lactic acid. As a consequence, it can be hypothesized that *C. diazotrophica* contributes to its production. Strong positive correlations were also observed with volatile compounds such as hexan-1-ol, methyl hexanoate and phenol, however, it must be stressed they were weaker than the ones observed with yeast species. In addition, a phylogenetic analysis performed on OTU representative sequences of the metabarcoding dataset and those of type species from the *Celerinatantimonas* genus revealed that these OTUs formed a separate clade strongly supported by bootstrap values (data not shown). Based on these elements, more work is undeniably needed to better characterize and determine the role of this bacterium during olive fermentations, especially regarding lactic acid production. Unfortunately, *C. diazotrophica* growth conditions make it difficult to cultivate. In fact, its presence during fermentation was actually overlooked by the culture-dependent analyses used in this study. Besides this species, which population could not be quantified, bacterial populations were very low for the entire fermentation.

Most surprisingly, LAB populations were scarce throughout the fermentation. This is an unusual finding as it is well established that spontaneous black olive fermentations rely on both LAB and yeasts and the dominance of one group over the other often depends on the fermentation stage (Bleve et al., 2014). In our case, LAB were detected from olive samples in the first days of fermentation (fresh fruit) but did not persist throughout fermentation. Both culture-dependent and -independent analyses concurred to this conclusion. Population levels were below the detection limits and only three species, *L. lactis*, *L. mesenteroides* and *L. paraplantarum*, were detected in low abundances (<0.5% of total species). LAB presence and dominance are affected by high phenolic content and salt concentration above 8% (Tassou et al., 2002). Consequently, the high levels of oleuropein during most of the fermentation and the traditional PDO specifications using 10% salt brine for Nyons olives most probably explain these findings. Moreover, correlation analyses did not reveal any significant correlation between these LAB species and fermentation markers such as lactic acid concentration and pH decrease. These results suggest a limited input in Nyons olive fermentation.

Yeasts were thus clearly identified as the microorganisms having the strongest input during fermentation. In this study, a core mycobiota was identified and included five species, *W. anomalus*, *C. nyonsensis*, *Z. mrakii*, *C. boidinii* and *P. membranifaciens*. All of these species have been individually identified in diverse table olive preparations (Coton et al., 2006; Nisiotou et al., 2010; Bautista-Gallego et al., 2011; Alves et al., 2012). In our study, they were systematically present in all fermentations although their respective dominance fluctuated according to fermentation stage. The ability of yeast to enhance fermented olive final product aromas has often been pointed out (Arroyo-López et al., 2008; Aponte et al., 2010; Arroyo López et al., 2012) and this was confirmed in our study as strong correlations were determined between yeast composition and volatile changes during fermentation. In summary, the Nyons olive fermentation could be dissociated into four phases. After an initial and fast decrease of plant-associated fungal and bacterial populations (phase I – day 1 to 8), the second fermentation phase (21–120 days) was dominated by *W. anomalus and C. nyonsensis* for fungi and *C. diazotrophica* for bacteria. As previously stated, it is highly probable that their main role during this phase is related to the pH decrease and malic acid production as no strong correlation was observed for these yeasts with any volatile compounds. The third phase of fermentation, between 120 and 183 days, was characterized by an increase in most acids, especially citric, lactic, succinic and acetic acids. This was linked to an increased abundance of *Z. mrakii*, *P. membranifaciens* and *C. boidinii.* as well as the highest abundances of 2-phenylethanol, methyl propanoate and octanoate esters. Both ester classes were previously linked to table olives (Tufariello et al., 2015) and positively impacted aroma by their characteristic sweet-fruity and acidic notes while 2-phenylethanol is characterized by a rose-like aroma and was shown to result from L-phenylalanine metabolism by yeast (Eshkol et al., 2009). The last months of fermentation (phase IV, between days 267 and 482) were correlated to an increase in ester and aromatic alcohol abundances. At this time, *P. membranifaciens* dominated and *C. boidinii* abundance further increased. These two species had the strongest correlations with numerous volatile compounds. Among them, ethyl propanoate, ethyl 2-methylbutanoate, phenylmethanol, 3-methylbutyl acetate and acetic acid were clearly identified and are considered as major aroma-active compounds in black table olives (Selli et al., 2018), thus supporting the key active roles of the two species.

These microbial dynamics were similar in the twelve monitored batches regardless of fruit maturity (two harvest periods) and cultivation practices (organic and conventional olives), thus highlighting the hardiness of the microbial fermentation process. No major differences were observed regarding aroma profiles between olive types so the use of conventionally or organically grown olives does not appear to favor any specific aroma compounds of the final product. Concerning harvest period, some differences were noticed. Acid content was higher for early compared to late harvest fermentations, whereas some volatile compound contents were higher in the late harvest fermentations. However, volatile compound differences could not be linked to any specific species presence or abundances. The observed differences in volatiles thus most likely derived from the fruit composition rather than microbial community variations. For instance, aldehydes are commonly retrieved in olive fruits as a result of endogenous lipid oxidation and their content was shown to increase with fruit maturity (Salas et al., 1999). As a result, the higher aldehyde content in the first weeks of late harvest fermentation is not surprising. Furthermore, if microbial community variations did not impact the fermentation process, as final olive characteristics were similar, a possible explanation would be that the distinctive microorganisms shared the same functionalities. An element to reinforce this hypothesis is that the aroma profile changes we observed for Nyons table olives were close to the ones recently described for Kalamata natural olive fermentations, although species diversity and dynamics differed as *S. cerevisiae*, *W. anomalus* and LAB were the drivers of the fermentation (Bleve et al., 2015). More data are clearly needed to corroborate this hypothesis as only a little number of studies are currently available to compare findings.

Overall, results of the present study described the microbial dynamics of Nyons table olive natural fermentations and how the microbiota shapes the final fermented olive characteristics. The polyphasic approach implemented, that merged microbiological and biochemical data with metabarcoding analyses, allowed us to have an in-depth understanding about microbial species succession and potential functional roles during Nyons table olive fermentations and determine the key drivers of this process. Our data revealed complex fungal species diversity. The main microbial drivers could also be correlated with certain specificities of these olives and constituted a first step to better understand microbial functionalities involved in this fermentation process. With regards to the culture-dependent approach, it mainly identified the dominant fungal species but also some sub-dominant ones (*P. carsonii* and *S. etchellsii*) and provided information about viable microorganisms, thus confirming trends observed using metabarcoding. Moreover, a large working collection of isolates, belonging to both dominant and subdominant species and presenting interesting biochemical profiles, was established from different time points throughout the fermentation. These microbial resources are of great interest to further characterize the major fermentation drivers, assess their technological features and better understand their contribution during Nyons olive fermentation.

## Data Availability Statement

The datasets presented in this study can be found in online repositories. The names of the repository/repositories and accession number(s) can be found below: https://www.ebi.ac.uk/metagenomics/, PRJEB39897; https://www.ebi.ac.uk/metagenomics/, PRJEB39898.

## Author Contributions

MC, JM, S-MD, and HF obtained the funding and supervised the study, edited and proofread the manuscript. MC, JM, and MP designed the experiments. MP performed the experiments and analyzed all the data, drafted the manuscript. AP provided technical assistance for DNA extractions for metabarcoding and culture-dependent experiments and CL with microbiological analyses. MC, GV, and EP performed HPLC and LC-MS data acquisition and analyses while AT and M-BM assisted with GC-MS analyses. JM and HF provided technical support for metabarcoding analyses. All authors contributed to the article and approved the present version.

## Conflict of Interest

The authors declare that the research was conducted in the absence of any commercial or financial relationships that could be construed as a potential conflict of interest.

## Footnotes

1 http://www.geneious.com

2 http://www.ncbi.nlm.nih.gov/BLAST
