# Supplementary material for: Deciphering Microbial Community Dynamics and Biochemical Changes During Nyons Black Olive Natural Fermentations
Source: Front Microbiol. 2020 Oct 8;11:586614. doi: 10.3389/fmicb.2020.586614 (PMC7578400; doi:10.3389/fmicb.2020.586614)
Supplement: Supplementary file 1 [file Data_Sheet_1.PDF]

Supplementary Material

1. Supplementary Figures

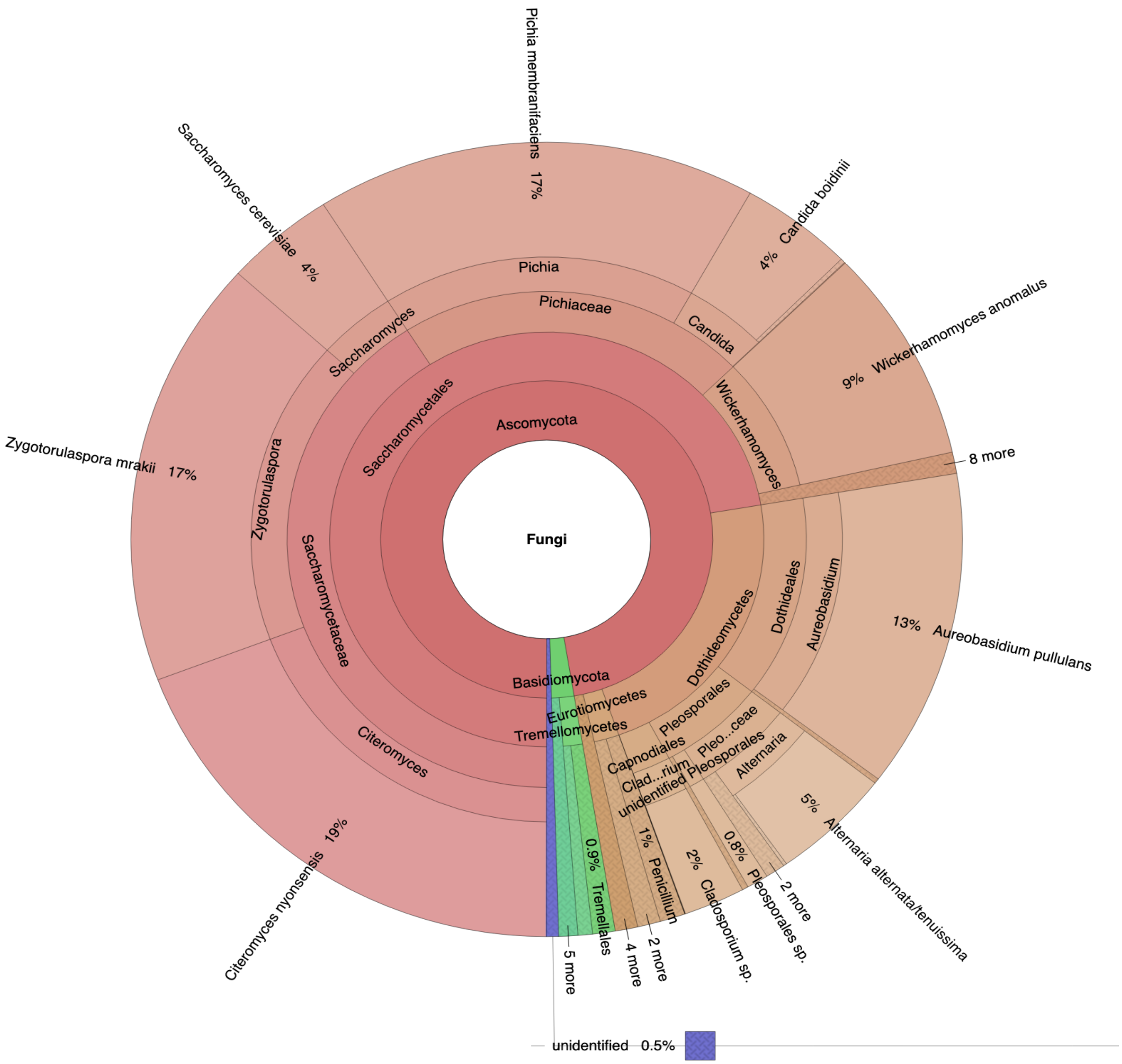

Supplementary Figure S1. Global fungal community composition of Nyons table olives based on ITS2 metabarcoding sequencing data.

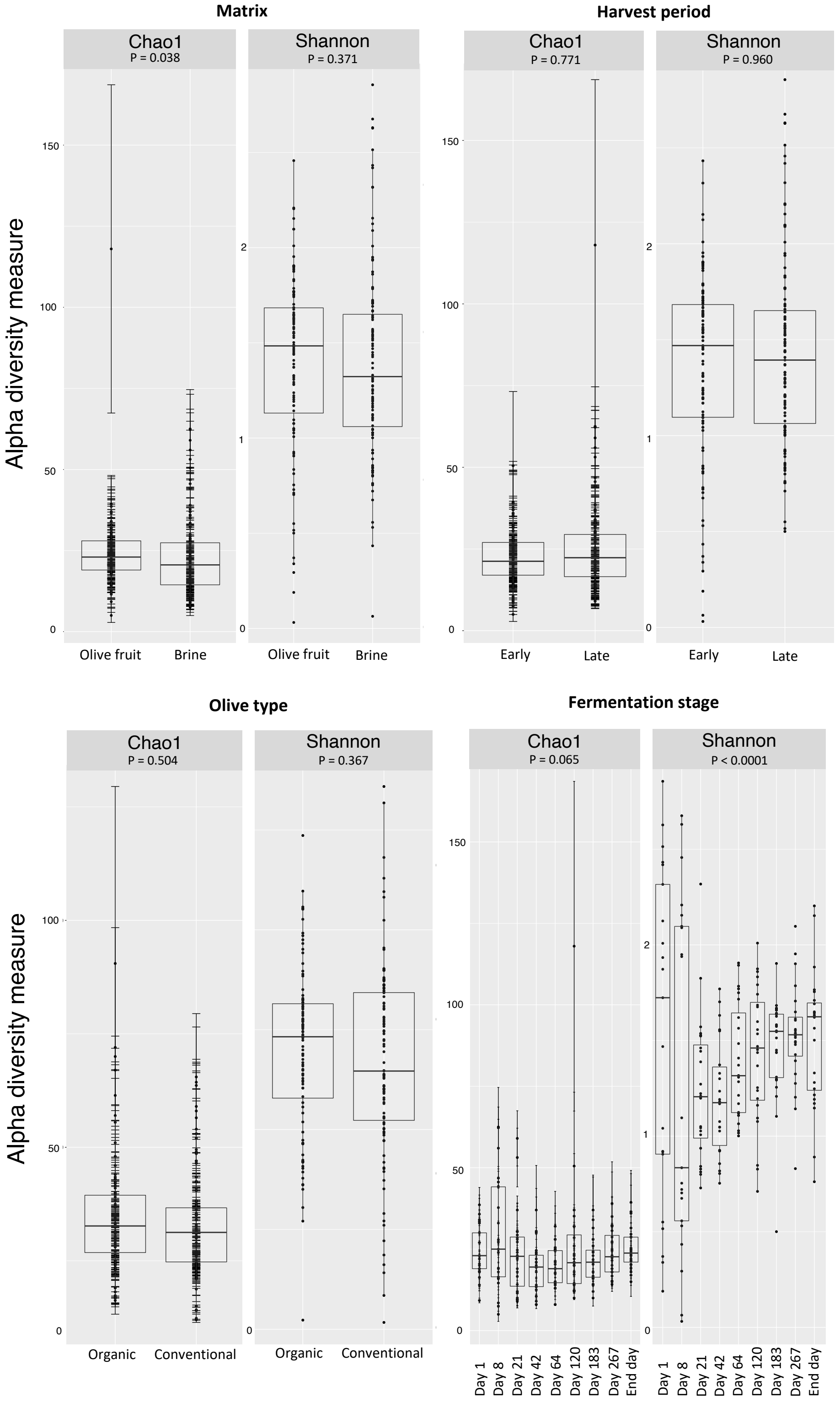

Supplementary Figure S2. Alpha-diversity indexes of fungal communities based on ITS2 metabarcoding sequencing data. Pvalues based on Kruskal-Wallis test (threshold 0.05) are given under each index.

A

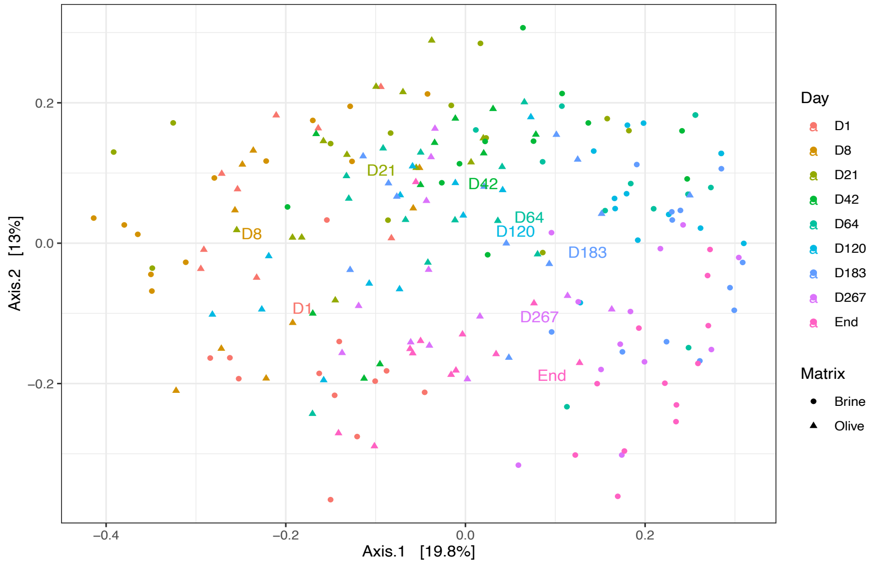

Adonis test

Stage	Matrix
P = 0.001	P =0.001
R^2 = 0.26	R^2 = 0.05

B

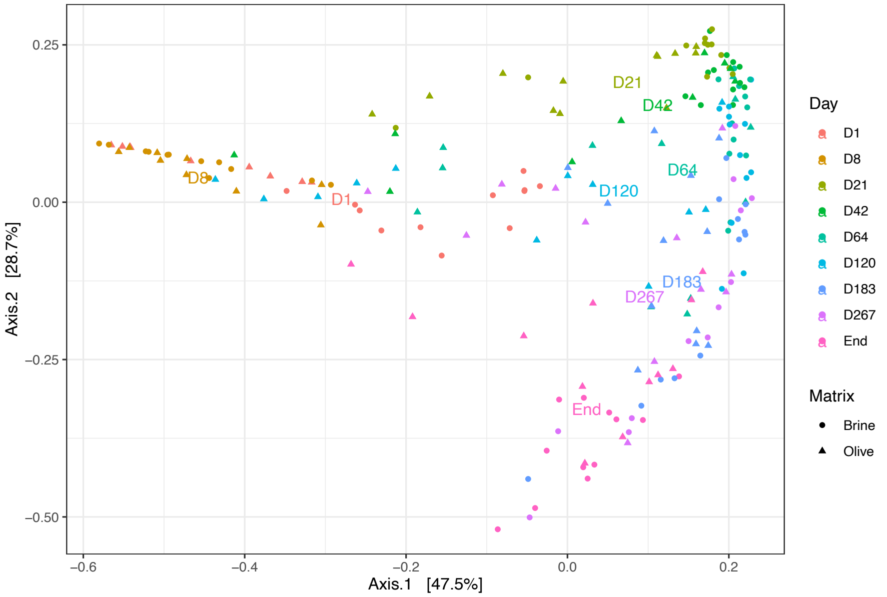

Adonis test

Stage	Matrix
P = 0.001	P =0.001
R^2 = 0.54	R^2 = 0.02

Supplementary Figure S3. Principal Coordinate analysis (PCoA) based on (A) unweighted Unifrac and (B) weighted Unifrac distances between fungal communities. P and R^2 values were obtained using Adonis test with 999 permutations for significance testing and partitioning of variance according to fermentation stage and matrix (Brine VS Olive) factors.

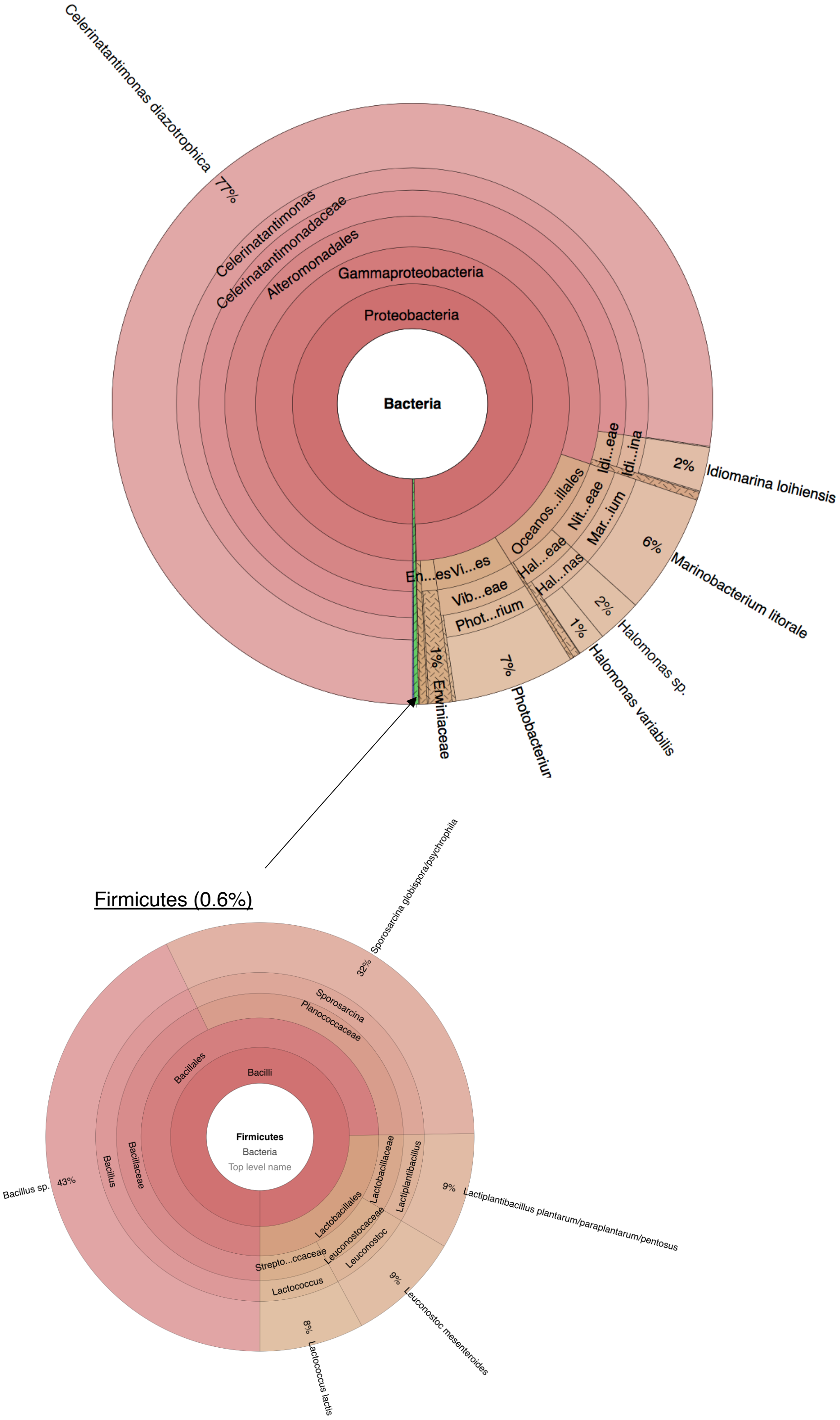

Supplementary Figure S4. Global bacterial community composition of Nyons table olives based on V3-V4 region of rRNA gene metabarcoding sequencing data.

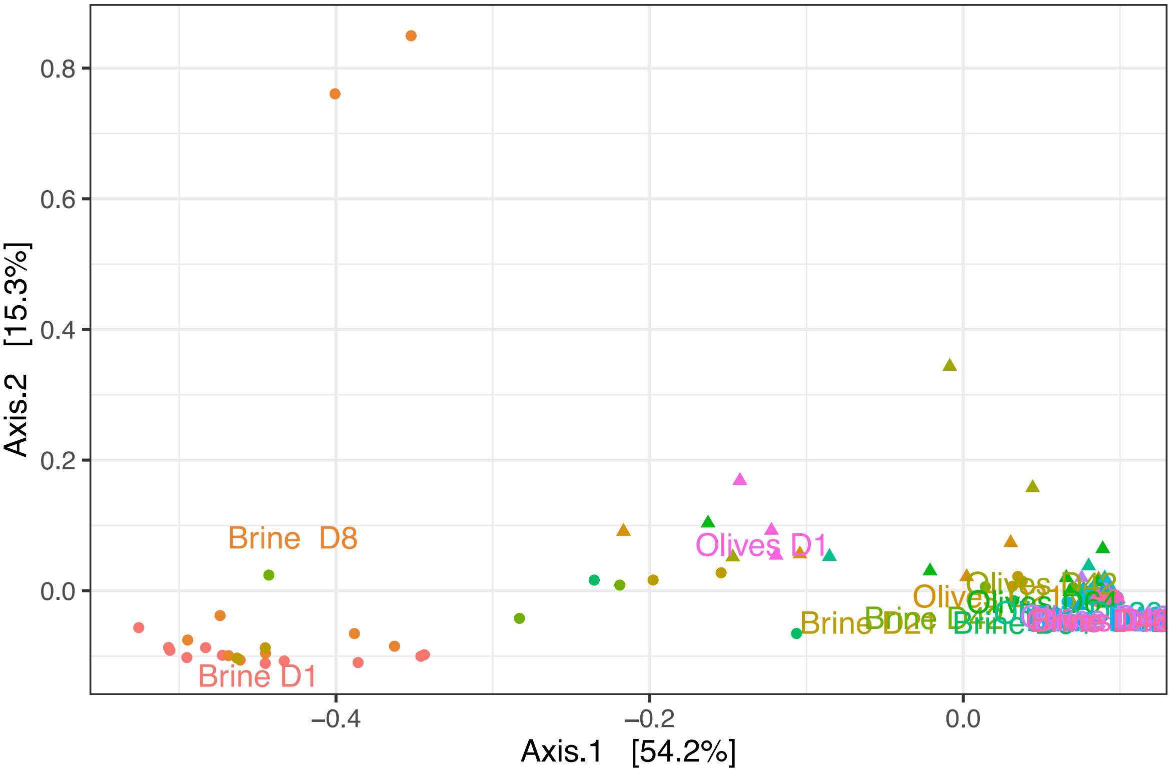

<u>Adonis test</u>

Stage	Matrix
P = 0.001	P =0.001
R^2 = 0.26	R^2 = 0.03

Supplementary Figure S5. Principal Coordinate analysis (PCoA) based on unweighted Unifrac distances between bacterial communities. P and R^2 values were obtained using Adonis test with 999 permutations for significance testing and partitioning of variance according to fermentation stage and matrix (Brine VS Olive) factors.

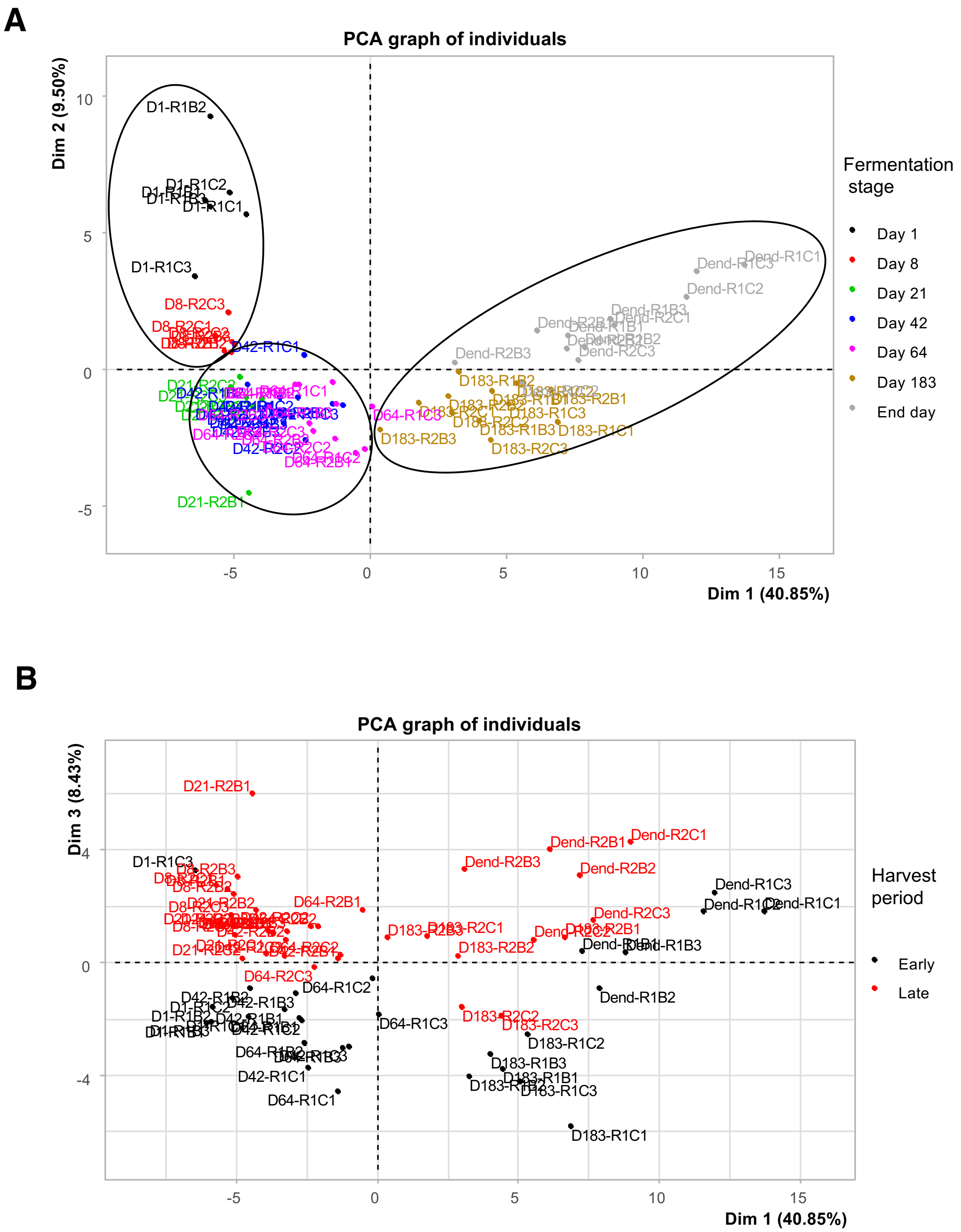

Supplementary Figure S6. PCA plots of volatile compounds from the 67 volatile compounds detected during Nyons table olive fermentations. The first 3 dimensions explained 58.78% of the total variance. (A) PCA score plot showing principal component 1 (Dim1) versus 2 (Dim2); (B) PCA score plot showing principal component 1 (Dim1) versus 3 (Dim3).

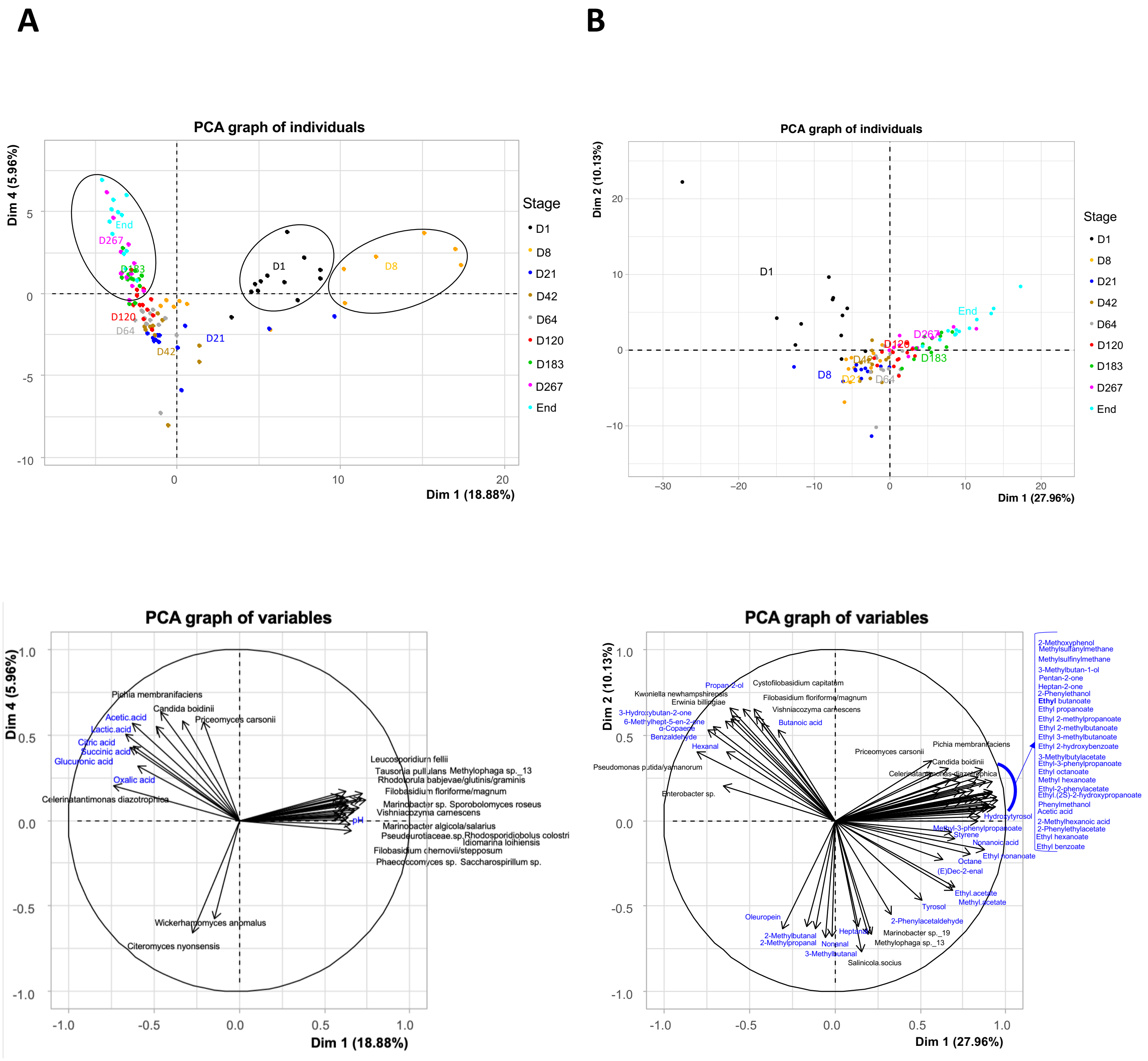

Supplementary Figure S7. Principal component analyses of microbial culture-independent and biochemical data (acids and volatile compounds in blue) throughout fermentation in brine **(A)** and in olive fruit(**B**). For both matrices, individual and variable plots are presented with sampling times considered as individuals. Variable plots considered species abundances (black) and organics acid concentration for brine and volatile compounds for olive fruit (blue).

2. Supplementary tables

Supplementary Table S1. Primers used in this study

Primer	Sequence	Target	Reference
ITS4	5’-TCCTCCGCTTATTGATATGC-3’	ITS region	White et al. (1990)
ITS5	5’-GGAAGTAAAAGTCGTAACAAGG-3’		
Bt2a	5’-GGTAACCAAATCGGTGCTGCTTTC-3’	β-tubulin gene	Glass and Donaldson (1995)
Bt2b	5’-ACCCTCAGTGTAGTGACCCTTGGC-3’		
NL1	5’-GCATATCAATAAGCGGAGGAAAAG-3’	D1-D2 domain	Kurtzman and Robnett (1997)
NL4	5’-GGTCCGTGTTTCAAGACGG-3’		
fD1	5’-CCGAATTCGTCGACAACAGAGTTTGATCCTGGCTCAG-3’	16S rRNA gene	Weisburg et al. (1991)
rP2	5’-CCCGGGATCCAAGCTTACGGCTACCTTGTTACGACTT-3’		
TuflparacF	5’-TCCGGGAACTGCTCAGC-3’	tuf gene	modified from Achilleos and Berthier (2013)
TuflparacR	5’-TGTTTCACGAACAGGTG-3’		
S-D-Bact-0341-b-S-17	5’-CCTACGGGNGGCWGCAG-3’	V3-V4 region of 16S rDNA gene	Klindworth et al. (2013)
S-D-Bact-0785-a-A-21	5’-GACTACHVGGGTATCTAATCC-3’		
ITS3f	5’-GCATCGATGAAGAACGCAGC-3’	ITS2 region	Toju et al. (2012)
ITS4_Kyo1	5’-TCCTCCGCTTWTTGWTWTGC-3’		

Supplementary Table S2. Identification based on 16S rRNA gene sequencing of bacteria isolated from Nyons table olives during fermentation

**Taxonomy**						**Fermentation***	**Number of isolates**			
**Phylum**	**Class**	**Order**	**Family**	**Genus**	**Species**		**Day 1**	**Day 64**	**Day 183**	**Total**
Firmicutes	Bacilli	Bacillales	Paenibacillaceae	*Paenibacillus*	*Paenibacillus* sp.	R1-Orga	0	1	0	1
Firmicutes	Bacilli	Bacillales	Staphylococaceae	*Staphylococcus*	*Staphylococcus epidermidis*	R1-Orga ; R1-Conv ; R2-Orga ; R2-Conv	0	0	1	1
Firmicutes	Bacilli	Bacillales	Staphylococaceae	*Staphylococcus*	*Staphylococcus warneri*	R1-Orga ; R1-Conv ; R2-Orga ; R2-Conv	2	2	2	6
Firmicutes	Bacilli	Bacillales	Staphylococaceae	*Staphylococcus*	*Staphylococcus hominis*	R1-Orga ; R2-Conv	0	2	1	3
Firmicutes	Bacilli	Bacillales	Staphylococaceae	*Staphylococcus*	*Staphylococcus* sp.	R2-Conv	0	0	1	1
Firmicutes	Bacilli	Bacillales	Staphylococaceae	*Staphylococcus*	*Staphylococcus capitis*	R1-Conv	0	0	1	1
Firmicutes	Bacilli	Lactobacillales	Lactobacillaceae	*Lacticaseibacillus*	*Lacticaseibacillus paracasei*	R2-Orga	1	0	0	1
Firmicutes	Bacilli	Lactobacillales	Leuconostocaceae	*Leuconostoc*	*Leuconostoc mesenteroides*	R1-Orga	2	0	0	2
Firmicutes	Bacilli	Lactobacillales	Streptococcaceae	*Lactococcus*	*Lactococcus lactis*	R2-Orga	4	0	0	4
Proteobacteria	Betaproteobacteria	Burkholderiales	Burkholderiaceae	*Ralstonia*	*Ralstonia* sp.	R1-Conv ; R2-Corga ; R2-Conv	0	4	0	4
Proteobacteria	Gammaproteobacteria	Enterobacterakes	Enterobacteriaceae	*Raoultella*	*Raoultella terrigena*	R1-Orga ; R2-Orga	4	0	0	4
Proteobacteria	Betaproteobacteria	Burkholderiales	Burkholderiaceae	*Ralstonia*	*Ralstonia* sp.	R1-Conv ; R2-Corga ; R2-Conv	0	4	0	4
Proteobacteria	Gammaproteobacteria	Oceanospirillales	Halomonadaceae	*Halomonas*	*Halomonas alkaliantarctica*	R1-Conv ; R2-Conv	4	0	0	4

*R1-Orga: Early harvest-Organic olives; R1-Conv: Early harvest-Conventional olives ; R2-Orga: Late harvest-Organic olives; R2-Orga: Late harvest-Conventional olives.

Supplementary Table S3. Organic acid quantification during Nyons table olive fermentations using LCMS and HPLC. Mean values of 3 replicates ± standard deviation are given. Means with different letters in each column and each fermentation are significantly different (Tukey's test p<0.05).

Sampling day	pH	Total acid (mM)	Acetic acid	Citric acid	Gluconic acid	Glucuronic acid	Lactic acid	Malic acid	Oxalic acid	Succinic acid
**pvalue**										
Harvest	-	-	0.16	**< 0.0001**	0.911	**0.002**	**0.006**	**< 0.0001**	0.794	**< 0.0001**
Olive type	-	-	0.39	0.14	0.149	0.237	**< 0.0001**	0.74	**0.016**	0.279
Stage	-	-	**< 0.0001**	**< 0.0001**	**< 0.0001**	**< 0.0001**	**< 0.0001**	**< 0.0001**	**< 0.0001**	**< 0.0001**
**R1-Orga**										
day 1	7.58 ± 0.04	0	0 a	0 a	0 a	0 a	0 a	0 a	0 a	0 a
day 21	4.95 ± 0.11	12.90	47.1 ± 9.8ab	1052.6 ± 181.8ab	46.3 ± 13.2ab	20.5 ± 7.2ab	113.8 ± 152.5a	167.6 ± 160.3a	177.4 ± 47.4ab	213.4 ± 183.1ab
day 42	4.68 ± 0.08	22.00	291.2 ± 140.3ab	1495.9 ± 451.3abc	46.7 ± 19.4bc	28.5 ± 12.1ab	196.2 ± 141.7ab	110.2 ± 127.1a	246.2 ± 108ab	383.2 ± 135.6bc
day 64	4.66 ± 0.08	38.38	708.7 ± 201.8ab	2307.6 ± 777.7bc	64.5 ± 24.9bcd	39.7 ± 19.2abc	340.3 ± 163.5ab	175.3 ± 119a	410.8 ± 170.9b	518.2 ± 100.9bcd
day 120	4.52 ± 0.03	52.52	988.5 ± 182.2ab	2829 ± 581.3cd	65.2 ± 12.4cde	60.2 ± 18.2bcd	783.8 ± 196.8ab	185.8 ± 182.3a	486.6 ± 74.6bc	614.1 ± 102.8cde
day 183	4.56 ± 0.05	77.05	1417.5 ± 133.2bc	4113.6 ± 634.7d	53.1 ± 8.8cde	81.5 ± 22.7cd	1275 ± 429.7ab	74.7 ± 37.9a	829.4 ± 114.6cd	876.5 ± 183.7cde
day 267	4.36 ± 0.02	103.07	2613.1 ± 1340.5c	4368.6 ± 848.3de	55.1 ± 5.5cde	82.9 ± 17cd	1583.5 ± 724b	106.4 ± 76.2a	1012.7 ± 204.2d	766.4 ± 110.8cde
day 482	4.47 ± 0.04	127.69	2719.4 ± 119.1c	6015.9 ± 833.5e	44 ± 5.5e	94.6 ± 19.4d	3020.8 ± 1110.1c	110.8 ± 48.2a	960.4 ± 232.4d	632.3 ± 97e
**R1-Conv**										
day 1	7.67 ± 0.02	0.00	0 a	0 a	0 a	0 a	0 a	0 a	0 a	0 a
day 21	4.75 ± 0.11	13.16	53.1 ± 5.2a	1119 ± 133.2ab	29.3 ± 0.5ab	15.6 ± 0.2ab	160.6 ± 146.9a	284 ± 15.7b	193.1 ± 124.2a	21.1 ± 12a
day 42	4.57 ± 0.04	33.36	751.2 ± 172.6ab	1986.5 ± 483.9bc	45.7 ± 14.2b	23.3 ± 4.5ab	221.7 ± 22.4a	393.5 ± 67.3b	373.1 ± 157.9ab	72.6 ± 6.9a
day 64	4.56 ± 0.03	39.77	877.3 ± 242.6ab	1987.9 ± 540.5bc	39.2 ± 19.4b	24.6 ± 8.2ab	613.8 ± 75.5a	414.3 ± 81.6b	326.1 ± 132.2ab	113.6 ± 44.7a
day 120	4.49 ± 0.06	60.75	1293.8 ± 601.1bc	3538.1 ± 524.2cd	41.8 ± 14b	47.1 ± 15.9abc	589 ± 92.7a	15.3 ± 26.2a	691.3 ± 55.2bc	708.5 ± 96.2b
day 183	4.51 ± 0.02	92.97	1557.4 ± 322.8bcd	4298.2 ± 864.8de	47.7 ± 10.7b	58.7 ± 25.6bc	2353.1 ± 726.1b	53.7 ± 26.3a	1020.1 ± 173.1c	740.2 ± 197.2b
day 267	4.37 ± 0.05	124.74	1974.5 ± 520.9cd	5443.3 ± 1091e	41.8 ± 14b	62.5 ± 29.4bc	4192 ± 313.2c	60.7 ± 39.3a	929.5 ± 232.5c	670.8 ± 127.2b
day 482	4.46 ± 0.06	148.98	2274 ± 339.9d	5901 ± 635.3e	43.8 ± 9.8b	87.4 ± 36.8c	5736.4 ± 451.3d	ND	883.9 ± 239.7c	654.6 ± 124.2b
**R2-Orga**										
day 1	7.42 ± 0.02	0.00	0 a	0 a	0 a	0 a	0 a	0 a	0 a	0 a

day 8	5.26 ± 0.02	5.72	0 a	706 ± 64.8ab	49 ± 0.7a	5.7 ± 0.9a	0	228.5 ± 15.3a	0 a	7.6 ± 1.9a
day 21	4.91 ± 0.21	14.10	176.5 ± 352.7ab	1166.2 ± 658.1ab	52.8 ± 20.3a	21.1 ± 3.4a	0 ± 0a	332.3 ± 75.2a	167.3 ± 151.7a	44 ± 26.7a
day 42	4.47 ± 0.06	46.09	1093.9 ± 150.5abc	1811.1 ± 681.5ab	29.7 ± 8.3a	25.4 ± 8a	828.8 ± 257.6ab	389.9 ± 111a	422.6 ± 413.4a	161 ± 38.8a
day 64	4.4 ± 0.03	46.01	913.2 ± 705.6abcd	2075.5 ± 1050.3ab	18.5 ± 6.4ab	22.2 ± 11.8a	990.4 ± 437.4ab	372.3 ± 181a	422 ± 121.9a	157.2 ± 43.5ab
day 120	4.38 ± 0.03	34.16	777.9 ± 582.5bcde	1598 ± 841.5ab	24.7 ± 10.4abc	32.6 ± 19.6a	427.5 ± 207.9ab	382.7 ± 226.8a	301.9 ± 203.1a	194.3 ± 88.2abc
day 183	4.39 ± 0.03	69.21	2098.1 ± 247.6cde	2313.7 ± 1063.4ab	27.2 ± 13.3bc	37.5 ± 24a	942.4 ± 371.3a	317 ± 304.7a	576.7 ± 460.1a	315.2 ± 109.6bc
day 267	4.13 ± 0.06	68.56	1488.1 ± 404.5de	3288.6 ± 1481.4b	33 ± 19.8bc	44 ± 28.5a	1223 ± 297.5a	350.2 ± 307.9a	670.5 ± 459a	310.9 ± 93bc
day 462	4.35 ± 0.08	74.20	1828.2 ± 307.5e	3526.5 ± 1765.7b	46.1 ± 23.2c	53.4 ± 37.3a	836.5 ± 613.8a	295.2 ± 192.9a	903.4 ± 891.9a	397.6 ± 127.9c
**R2-Conv**										
day 1	7.44 ± 0.04	0.00	0 a	0 a	0 a	0a	0a	0a	0 a	0 a
day 8	5.22 ± 0.02	4.10	0 a	464.2 ± 53.2ab	35 ± 6.3ab	3.8 ± 0.7ab	0 ± 0a	198.5 ± 47.4a	0 a	0.9 ± 0.4a
day 21	5.1 ± 0.01	12.88	0 a	826.7 ± 157.4abc	83.6 ± 29.6cd	12.7 ± 3.8abc	56.1 ± 52.3a	318.6 ± 98.3a	434.5 ± 173ab	30.4 ± 11.7a
day 42	4.83 ± 0.12	33.67	182.9 ± 28.2a	1593.2 ± 391bcd	113 ± 23.8d	25.2 ± 6.8abcd	538.6 ± 113.8b	418.5 ± 148.1a	1017.6 ± 467.1ab	144.9 ± 38.6a
day 64	4.69 ± 0.05	37.10	191 ± 38.3a	2085.2 ± 639.2cde	62.5 ± 15.1bc	33.1 ± 13.6bcde	1126.4 ± 281c	467.9 ± 156.8a	445.9 ± 282abc	192.9 ± 7.7a
day 120	4.54 ± 0.03	47.69	749.9 ± 440.3ab	2190 ± 354.3de	41 ± 14.3abc	33.6 ± 7.1bcde	1106.3 ± 171.1c	352.8 ± 305.5a	526.8 ± 94.9bc	313.6 ± 194.2ab
day 183	4.5 ± 0.04	78.86	1893 ± 746bc	3159.3 ± 722.1ef	35.4 ± 3ab	41.7 ± 11cde	1390.1 ± 110.5cd	164.5 ± 282.6a	781.6 ± 374.2bc	609.7 ± 281.6bc
day 267	4.28 ± 0.06	92.45	2279.5 ± 943.8bc	3563.4 ± 579f	48.7 ± 11.1bc	54.6 ± 14.3de	1668.9 ± 95.3d	68.3 ± 52a	919.2 ± 273bc	728.6 ± 130.1bc
day 462	4.39 ± 0.07	100.58	1752.5 ± 16.2c	4152 ± 619.2f	46.8 ± 7.1bc	59.6 ± 19.8e	2629.6 ± 331.1e	133.7 ± 94.1a	1208.7 ± 119c	664.5 ± 140.9c

R1-Orga: Early harvest-Organic olives; R1-Conv: Early harvest-Conventional olives ; R2-Orga: Late harvest-Organic olives; R2-Orga: Late harvest-Conventional olives.

Supplementary Table S4. Volatile compounds identified during Nyons table olive fermentation using HS-GC-MS

Family	Compound	N° Pubchem	LRI[a]	EIC (m/z)	Identification[b]	Pvalue[c]			Group[d]	Odor descriptor[e]
						Harvest	Olive type	Stage		
Alcohols	Ethanol	702	931	45	RI, DB, S	8.48E-02	1.00E-01	9.31E-01	-	Alcoholic
	Hexan-1-ol	8103	1382	56	RI, DB	**3.95E-07**	**1.61E-09**	**9.54E-08**	III	Green, fruity, pear
	3-Methylbutan-1-ol	31260	1222	55	RI, DB, S	3.13E-01	**1.37E-02**	**2.52E-21**	III	Fermented, fruity, pungent
	Phenylmethanol	244	-	108	DB	8.16E-02	7.59E-01	**4.05E-20**	III	Floral, rose, phenolic
	2-Phenylethanol	6054	-	91	DB	6.21E-01	7.61E-01	**1.15E-10**	II	Floral, sweet, rosey
	Propan-2-ol	3776	923	59	RI, DB	5.73E-01	9.90E-01	**2.29E-25**	I	Alcoholic
Aldehydes	Benzaldehyde	240	1518	106	RI, DB, S	**5.79E-03**	**2.05E-03**	**4.84E-30**	I	Almond
	(E)-Dec-2-enal	5283345	1638	83	RI, DB, S	7.92E-01	2.25E-01	**4.33E-19**	III	Fatty, green
	Hexanal	6184	1083	56	RI, DB	**7.09E-03**	5.43E-01	**2.12E-26**	I	Fatty, grenn, leafy
	Heptanal	8130	1185	70	RI, DB, S	1.18E-01	1.16E-01	**8.42E-04**	I	Tallowy, green
	2-Methylbutanal	7284	905	57	RI, DB, S	**2.43E-06**	1.29E-01	**3.54E-02**	II	Malty, nutty
	3-Methylbutanal	11552	909	44	RI, DB, S	**2.08E-03**	9.58E-01	2.58E-01	-	Malty
	2-Methylpropanal	6561	801	72	RI, DB, S	**6.97E-08**	6.87E-01	**2.91E-04**	I	Cooked-caramel
	Octanal	454	1284	84	RI, DB, S	5.01E-01	7.05E-02	**6.97E-04**	III	Fatty, green
	2-Phenylacetaldehyde	998	1642	91	RI, DB	6.15E-01	1.93E-01	**2.22E-02**	-	Honey, floral rose, fermented,
	4-Propylbenzaldehyde	120047	-	148	DB	**2.10E-02**	9.37E-02	**1.10E-15**	II	-
	Nonanal	31289	1392	98	RI, DB	**3.39E-02**	5.80E-01	**3.24E-03**	I	Fatty, waxy
Esters	Ethyl acetate	8857	876	61	RI, DB, S	8.79E-01	**1.53E-02**	**3.21E-19**	II	-
	Ethyl benzoate	7165	1664	105	RI, DB	8.23E-02	3.81E-01	**1.01E-20**	III	Mild, fruity, cooked
	Ethyl butanoate	7762	1033	71	RI, DB, S	3.56E-01	1.07E-01	**5.11E-25**	III	Sweet, fruity
	Ethyl hexanoate	31265	1233	88	RI, DB, S	**2.99E-03**	**1.44E-02**	**9.21E-08**	II	Sweet fruity
	Ethyl (E)-hex-2-enoate	5364778	1342	97	RI, DB	**3.86E-05**	**3.38E-03**	**9.14E-03**	II	Rum, fruity, juicy
	Ethyl 2-hydroxybenzoate	8365	-	120	DB	4.54E-01	4.97E-01	**3.00E-12**	III	Minty
	Ethyl (2S)-2-hydroxypropanoate	92831	1346	75	RI, DB	3.85E-01	1.31E-01	**3.79E-20**	III	Fruity
	Ethyl 2-methylbutanoate	24020	1050	57	RI, DB	**1.93E-02**	**7.51E-03**	**3.93E-21**	III	Sweet, fruity
	Ethyl 3-methylbutanoate	7945	1069	115	RI, DB, S	4.36E-01	1.79E-01	**1.45E-17**	III	Sweet, fruity
	Ethyl 3-methylbut-2-enoate	12516	1222	128	RI, DB	8.15E-02	**2.87E-02**	**8.05E-05**	III	-
	Ethyl 2-methylpropanoate	7342	956	101	RI, DB	1.98E-01	**3.25E-04**	**2.55E-20**	III	Etherial, fruity
	Ethyl octanoate	7799	1436	88	RI, DB	**1.51E-09**	9.56E-01	**5.13E-20**	II	Fruity, winey
	Ethyl nonanoate	31251	1537	101	RI, DB	**5.96E-03**	3.21E-01	**6.95E-17**	III	Fruity, rose, rum, wine
	Ethyl 2-phenylacetate	7590	1805	91	RI, DB	**2.47E-09**	7.71E-02	**4.77E-25**	II	Sweet, floral honey
	Ethyl 3-phenylpropanoate	16237	-	91	DB	**1.71E-03**	**2.11E-03**	**2.95E-23**	II	Floral, hyacinth, rose, honey
	Ethyl (E)-3-phenylprop-2-enoate	637758	-	131	DB	5.69E-01	3.53E-01	**4.23E-08**	II	Fruity, balsamic
	Ethyl propanoate	7749	947	102	RI, DB	6.90E-01	1.02E-01	**7.74E-16**	III	Fruity, winey
	Hexyl acetate	8908	1272	56	RI, DB	**2.23E-07**	**2.07E-04**	1.22E-01	II	Fresh, sweet, floral, green
	Methyl acetate	6584	814	74	RI, DB	6.82E-02	4.76E-01	**3.46E-18**	II	-
	3-Methylbutyl acetate	31276	1134	87	RI, DB	**3.88E-03**	5.17E-01	**8.37E-28**	III	fruity, green ripe
	Methyl hexanoate	7824	1188	74	RI, DB, S	**1.28E-07**	**8.41E-04**	**5.30E-15**	II	Fruity, pinapple, banana
	Methyl octanoate	8091	1389	74	RI, DB, S	**2.27E-10**	8.59E-01	**8.69E-13**	II	Apple, apricot
	Methyl 3-phenylpropanoate	7643	-	104	DB	**3.30E-02**	**6.00E-05**	**2.25E-22**	II	Honey, fruity, wine
	2-Methylpropyl acetate	8038	1012	43	RI, DB	2.90E-01	2.25E-01	**8.08E-04**	III	Banana, apple
	2-Phenylethyl acetate	7654	-	104	RI, DB	2.34E-01	2.17E-01	**8.93E-26**	III	Sweet, honey, floral rosy
	Texanol	6490	-	71	DB	3.15E-01	4.41E-01	**1.30E-02**	II	Plastic
Fatty acids	Acetic acid	176	1457	60	RI, DB, S	2.32E-01	1.40E-01	**1.47E-06**	III	Pungent, vinegar
	Butanoic acid	264	1637	73	RI, DB, S	1.30E-01	3.07E-01	**2.44E-07**	I	Acidic, buttery
	Hexanoic acid	8892	-	73	RI, DB	1.05E-01	**2.94E-03**	**2.03E-04**	I	Fruity, fatty sour
	4-Hydroxybutanoic acid	10413	1624	42	RI, DB	5.80E-01	**2.90E-03**	**1.56E-06**	II	-
	3-Methylbut-2-enoic acid	10931	-	82	RI, DB	5.75E-01	**5.73E-03**	**1.76E-07**	III	Green, phenolic
	2-Methylhexanoic acid	20653	1673	74	RI, DB	5.41E-01	9.60E-01	**8.38E-07**	III	Oily, creamy
	Nonanoic acid	8158	-	129	DB, S	2.42E-01	9.95E-01	**4.34E-06**	II	Fatty
	Octanoic acid	379	-	101	DB, S	3.91E-01	5.65E-01	**4.13E-05**	II	Oily, rancid, capric
	Propanoic acid	1032	1548	74	RI, DB, S	9.89E-01	4.60E-01	**2.15E-17**	III	Acidic, dairy, fruity
Ketones	Butane-2,3-dione	650	977	86	RI, DB, S	6.46E-01	**4.32E-02**	**4.97E-02**	-	Buttery
	Heptan-2-one	8051	1182	58	RI, DB, S	**1.64E-02**	9.56E-02	6.32E-02	-	Fruity, green banana
	3-Hydroxybutan-2-one	179	1279	88	RI, DB, S	3.84E-01	9.80E-01	**5.02E-12**	I	Sour milk
	6-Methylhept-5-en-2-one	9862	1334	108	RI, DB	7.65E-01	1.20E-01	**6.53E-07**	I	Green, citrus
	Pentan-2-one	7895	967	86	RI, DB	**9.08E-03**	5.53E-01	**4.59E-17**	III	-
Phenols	1,2-Dimethoxybenzene	7043	1727	138	RI, DB	8.70E-02	1.10E-01	**1.40E-20**	III	Spicy, vanilla
	4-Ethylphenol	31242	-	107	DB	**4.75E-02**	**4.95E-04**	**4.45E-11**	II	Phenolic, smoky
	2-Methoxyphenol	460	-	109	DB	7.61E-01	**3.54E-02**	**5.54E-21**	III	Phenolic, woody
	Phenol	996	-	94	DB	8.41E-01	**1.99E-02**	**8.17E-24**	III	Phenolic
Others	α-Copaene	19725	1483	119	RI, DB, S	**5.15E-03**	4.65E-01	**2.13E-04**	I	Woody, spicy, honey
	Methylsulfanylmethane	1068	736	62	RI, DB	1.74E-01	2.82E-01	**1.67E-06**	III	Sulfury
	Methylsulfinylmethane	679	1559	63	RI, DB	**7.28E-03**	1.79E-01	**2.99E-12**	III	Fatty, oily, salty
	(Methyltrisulfanyl)methane	19310	1368	126	RI, DB	6.69E-01	9.12E-01	9.74E-01	-	Sulfury
	Octane	356	786	85	RI, DB, S	1.16E-01	**1.42E-02**	**2.09E-24**	II	-
	Styrene	7501	1250	78	RI, DB	**8.00E-05**	**1.13E-02**	**5.62E-22**	II	Sweet, balsam, floral

a LRI = Linear Retention index on polar GC column - calculated based of retention time of n-alcane mixture injected in the column

b Identification performed based the following criteria: RI—comparison of LRI calculated with data published in the literature and in intern; DB—comparison of mass spectral data with those of NIST 2008 library ; and S—comparison of LRI with those of authentic standards injected in the same GC- MS system

c Pvalue obtained under ANOVA with 0.05 significance level

d Groups formed based on Hierachical clustering and heatmap results (see Figure 5)

e odor descriptor associated with the compounds on The goodscentscompany database database (http://www.thegoodscentscompany.com)
